# Why Is the Diversity of Tree Species in China’s Lowland Rainforests Higher than in Its Montane Rainforests?

**DOI:** 10.3390/plants14040505

**Published:** 2025-02-07

**Authors:** Tong Pang, Langxing Yuan, Yaqing Wei, Xin Wang, Ning Zhang, Kepeng Ji, Yuwu Li, Guoyu Lan

**Affiliations:** 1 Rubber Research Institute, Chinese Academy of Tropical Agricultural Sciences, Haikou 571101, China; pt1781857933@163.com (T.P.); hnweiyaqing@163.com (Y.W.); wxhzau273@163.com (X.W.); zning0105@163.com (N.Z.); hnjikepeng@163.com (K.J.); 2 College of Landscape Architecture and Forestry, Qingdao Agricultural University, Qingdao 266109, China; liyuwu@qau.edu.cn; 3 Hainan Danzhou Tropical Agro-Ecosystem National Observation and Research Station, Danzhou 571737, China; 4 Tropical Crops Genetic Resources Institute, Chinese Academy of Tropical Agricultural Sciences, Haikou 571101, China; yuanlangxing@163.com

**Keywords:** Hainan Island, lowland rainforest, montane rainforest, tree species diversity, niche overlap, distribution pattern

## Abstract

Despite extensive research on tree species diversity in tropical rainforests, the differences in diversity between lowland and montane rainforests, as well as the underlying mechanisms, remain unclear. This study utilizes tree inventory data from two dynamic monitoring sample plots, each with an area of 1 hm^2^, established in the lowland rainforest and montane rainforest regions of Diaoluo Mountain, Hainan Island. We analyzed the composition, diversity, spatial distribution patterns, and interspecific relationships within the tree communities. In total, 154 tree species with DBH > 3 cm were recorded in lowland rainforests, and 119 were recorded in montane rainforests, with lowland rainforests harboring 51 rare species compared to 40 rare species in montane rainforests. The distribution pattern of small trees (with DBH ≤ 7.5 cm) exhibited clustering at a small scale but random distribution at a larger scale. This phenomenon was more prevalent in tropical lowland rainforests, indicating that the negative density dependence effect is more pronounced in lowland rainforests compared to montane rainforests. Moreover, a higher proportion of negative associations and lower niche overlaps were observed in the lowland rainforest compared to the montane rainforest, suggesting that niche differentiation among tree species is more pronounced in the lowland rainforest. In conclusion, the more intense negative density dependence effect and niche differentiation are the primary factors contributing to the higher tree species diversity observed in lowland rainforests.

## 1. Introduction

Forest ecosystems are characterized by their remarkable biodiversity and represent the largest terrestrial ecosystems on Earth [1]. Tropical rainforests constitute one of the primary types of forest ecosystems. Although they occupy only 7% of the Earth’s land surface, they are home to over 60% of known animal and plant species, thereby playing a crucial role in the maintenance of global biodiversity [2,3]. Hainan Island is situated within a global biodiversity hotspot, featuring extensive areas of tropical rainforest. Tropical evergreen forests located below 900 m in elevation are classified as lowland rainforests, while those found between 600 and 1300 m are termed montane rainforests [4]. This classification closely aligns with the classical categorization of tropical rainforests proposed by Richards [2]. Mountain and lowland rainforests are the two most representative types of rainforests found in Hainan.

The mechanisms underlying biodiversity maintenance have consistently represented a core issue and challenge in ecological research [5,6,7]. Ecologists have formulated various hypotheses to elucidate the maintenance of biodiversity, among which the phenomenon of negative density dependence (NDD) is one of the widely accepted mechanisms [8,9,10,11]. Numerous studies have investigated the effects of negative density dependence [12,13,14,15]. Lan et al. [16,17,18,19,20] studied tree species diversity, spatial distribution patterns, and interspecific associations within tropical rainforests in Xishuangbanna, southwest China, finding that NDD is commonly present in these ecosystems, which indicates that the Janzen-Connell (JC) effect plays an important role in maintaining tree species diversity in tropical forest [21]. However, in a meta-analysis, scientists found that density-dependent and distance-dependent mortality rates vary significantly among genera and families, which suggests that the J-C effect may not be as ubiquitous as previously believed [12].

Niche differentiation refers to the phenomenon whereby species occupy distinct niches within ecosystems through various adaptation strategies and characteristics, thereby avoiding direct competition. As one of the pivotal mechanisms for maintaining community biodiversity, the theory of species coexistence grounded in niche differentiation has garnered significant attention. For instance, niche differentiation has been posited to elucidate how plant species exploit the fluctuating availability of resources during succession through trade-offs [22,23]. Hutchinson introduced the dimensions of the niche, including niche location, niche width, and overall niche morphology [24,25]. Among these, niche width is the most critical, as it reflects the needs and tolerances of the organism.

Despite numerous studies indicating that species diversity declines with increasing altitude [26], the differences in sampling scales and research methodologies among prior studies leave ambiguous whether montane rainforests or lowland rainforests exhibit greater species diversity in China [27]. It is particularly crucial to investigate the differences in diversity maintenance mechanisms between these two rainforest communities. Therefore, this study is based on tree species inventory data from two 1-hectare dynamic monitoring sample plots, established in the lowland rainforest and montane rainforest in Diaoluoshan, Hainan, to test the following hypotheses: (1) Lowland rainforests, characterized by lower elevation and higher temperatures, are associated with greater tree species diversity compared to montane rainforests, because species richness declines in accordance with increasing altitude [28,29]. (2) Along with the decease in altitude gradient, tree species diversity is expected to increase with greater negative density dependence of the same species [29,30]. Therefore, it is inferred that the NDD effect in lowland rainforests is stronger than that observed in montane rainforests. (3) Due to intense competition within the lowland rainforest community, it is posited that interspecific relationships are predominantly negatively correlated, characterized by low niche overlap and substantial niche differentiation [31,32,33,34]. The findings of this research aim to provide a foundational basis for biodiversity protection and ecological management of the rainforest in Hainan and are critically significant for establishing a comprehensive understanding of the ecological characteristics and conservation strategies of tropical rainforests on Hainan Island.

## 2. Materials and Methods

### 2.1. Study Site

**Figure 1:**
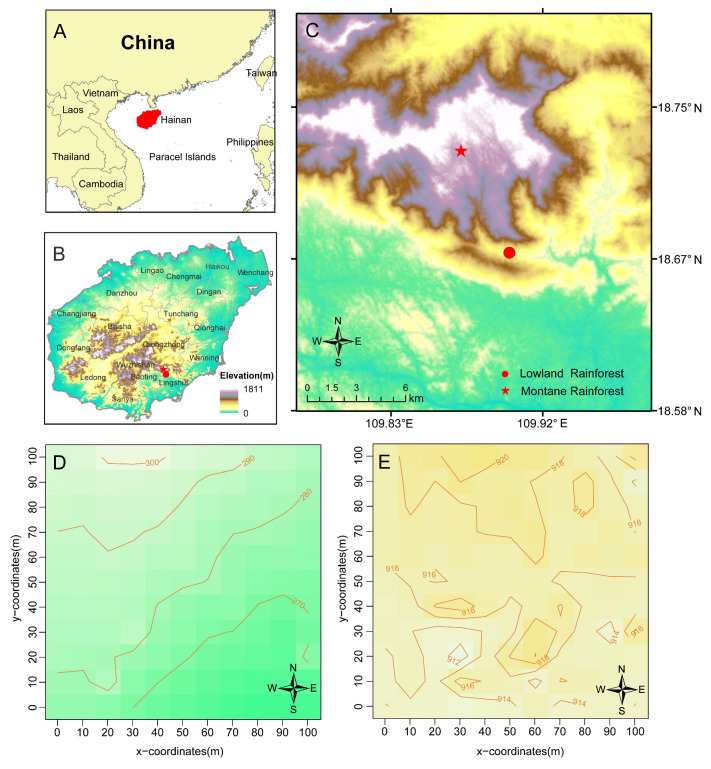
The locations (**A**–**C**) and topographic maps (**D**,**E**) for 1 hm^2^ dynamic monitoring plots of lowland rainforest and montane rainforest of Diaoluo Mountain. (**A**–**C**): The coordinate location of the dynamic monitoring plot. (**D**): A topographic map of the lowland rainforest dynamic monitoring plot. In the map, colors transitioning from green to yellow represent elevations increasing from low to high. (**E**): A topographic map of the montane rainforest dynamic monitoring plot. In the map, colors transitioning from yellow to brown also represent elevations increasing from low to high. Red solid circle: lowland rainforest; red solid star: montane rainforest.

Hainan Island is situated on the northern fringe of the tropics and is characterized by a warm, humid climate throughout the year, abundant rainfall, and dense tropical rainforests, with forest cover exceeding 50%. It constitutes a significant component of the global tropical rainforest ecosystem [35]. Hainan Island is not only a continental island rainforest with a concentrated distribution, excellent preservation, and extensive contiguous areas within China; it also serves as a unique repository of biodiversity, comprising a vast array of animal and plant species, as well as a globally recognized germplasm gene bank (Figure 1A,B). Furthermore, it is an essential region for the conservation of tropical biodiversity, both in China and worldwide. The Diaoluo mountain Nature Reserve, located in the southeastern part of Hainan Island, is not merely one of the significant tropical forest distribution areas in China; it also hosts the most developed tropical forest in the country, making it the area closest to equatorial rainforests [36].

### 2.2. Data Collection

In accordance with the protocol established for the 50 hm^2^ plot in Panama in 1980 [37,38,39,40], two dynamic monitoring plots, each with an area of 1 hm^2^, were established on Diaoluo Mountain, Hainan Island, from August 2023 to January 2024 (Figure 1C). Polyvinyl chloride (PVC) tubes were installed at 10 m intervals within the sample plots, and elevation data for each of these intervals were recorded. The elevation of the lowland rainforest plot ranges from 260 m to 302 m, while the montane rainforest plot is situated at an elevation of 913 m to 920 m (Figure 1D,E). Concurrently, the diameter at breast height (DBH) of each individual tree within the plots was measured, each tree was tagged with aluminum tags, and the relative coordinates of each tree in 10 × 10 m subplots were recorded. We measured the DBH, height, crown width, and other relevant information for all trees with a DBH ≥ 3 cm. Specimens of unidentified species were collected, photographed, and transported to the laboratory for identification. Tree species names were primarily determined based on the electronic version of the Chinese Flora. Detailed climatic information regarding the dynamic monitoring plots of Hainan’s mountain and its lowland rainforests is presented in Table S1.

### 2.3. Data Analysis

#### 2.3.1. Data Statistics

The dominant families (those containing more than five species), common families (containing more than two but fewer than five species), and singleton families (containing only one species) were subjected to statistical analysis. Additionally, the number and proportion of dominant species, common species, and rare species within the community were evaluated. Specifically, species with more than 50 individuals were classified as dominant species, species with between 2 and 50 individuals were categorized as common species, and species represented by only one individual throughout the entire plot were classified as rare species [41]. A species–area curve and species–sequence curves for the community were generated using the *vegan* package in the R environment [42], while species–abundance curves were fitted using a normal distribution. The importance values for each family and species within the community were calculated according to the formula for species importance value as follows: Important Value (*IV*) = Relative Frequency (*RF*) + Relative Dominance (*RD*) + Relative Density (*RA*) [43]. Furthermore, the importance value of a family is the sum of the importance values of all species within that family. To further analyze the dynamic changes in dominant species, the distribution of diameter classes for dominant species within the community was examined, with each diameter class representing a range of 2 cm, and a diameter class distribution map was constructed using R software (4.3.0).

#### 2.3.2. Spatial Distribution Pattern

To elucidate the spatial distribution patterns of dominant species of small trees, R software was employed to visualize the spatial distribution of these species within the sample plots, which included 22 tree species in the lowland rainforest and 15 tree species in the montane rainforest (DBH ≤ 7.5 cm, abundance ≥ 10). Subsequently, a distance-based spatial pattern analysis was conducted to determine whether the populations were randomly distributed, clustered, or uniformly distributed [44]. Point pattern analysis was performed using the univariate point pattern analysis method *g*(*r*). A Monte Carlo fitting test was utilized to calculate the upper and lower envelope traces, also known as confidence intervals. The number of fittings was set to 999, with a confidence level of 99%. If the test results are randomly distributed between the envelope traces, this indicates a significant aggregated distribution when exceeding the upper envelope trace, and a significant uniform distribution when falling below the lower envelope trace.

#### 2.3.3. Interspecific Association Analysis

The interspecific relationships within the community were examined using the bivariate point pattern analysis method *Gij*(*r*). A total of 55 pairs of dominant species were identified in the lowland rainforest, while 28 pairs were identified in the mountainous rainforest (individuals ≥ 50). The correlation between individuals (*i*) and (*j*) was analyzed by calculating the number of individuals (*j*) of different tree species located within a circular area centered on individual (*i*) with a radius (*r*), where (*i*) and (*j*) represent two arbitrary individuals of distinct dominant species within the community. Additionally, the Monte Carlo fitting test was employed to calculate the upper and lower envelope traces. If the test results indicate that the two dominant species fall within the envelope curves, then there is no correlation between them. The results indicated a significant positive correlation above the upper envelope trace and a significant negative correlation below the lower envelope trace. Univariate and bivariate analyses of point patterns were primarily conducted in the R programming language using software packages such as *spatial* [45], *spatstat* [46], *splancs* [47].

#### 2.3.4. Intraspecific Association Analysis

A bivariate point pattern analysis method *Gij*(*r*) was also used to reveal intraspecific associations between large trees (DBH ≥ 25 cm) and small trees (DBH ≤ 7.5 cm). A total of 21 tree species were analyzed in the lowland rainforest, while 11 tree species were analyzed in the montane rainforest.

#### 2.3.5. Niche Differentiation and Environmental Variables Analysis

A calculation of niche width and niche overlap was performed using the *spaa* package [48] in the R environment, and visualization was implemented using *ggplot2* [49] and *reshape2* [50]. The top 25 tree species were used to analyze niche overlap between lowland rainforest and montane rainforest. We calculate the niche overlap of species using the number of 10 m × 10 m subplots occupied by species within a one-hectare plot. And all species were used to calculate the niche width between lowland rainforest and montane rainforest. The niche width of species is calculated using the number of individuals distributed within a one-hectare plot. For the determination of soil physicochemical properties, we referred to previously adopted methods (see [51] for details).

## 3. Result Analysis

### 3.1. Species Composition and Diversity

**Figure 2:**
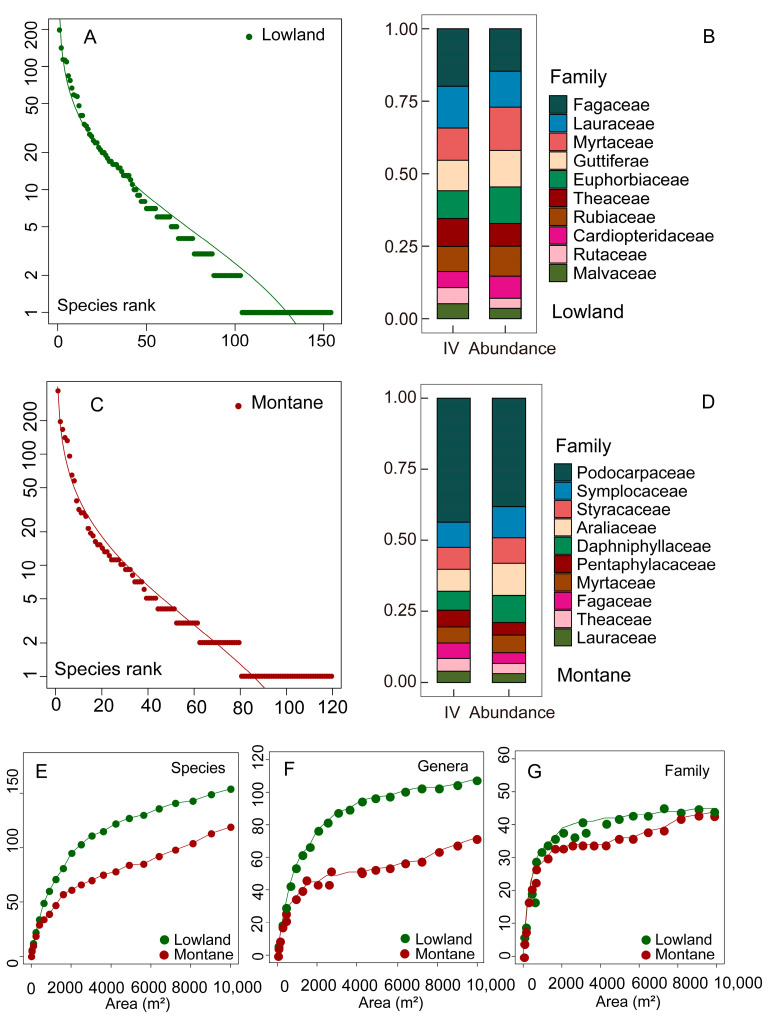
Species sequence curves (**A**,**C**); the top ten families with the highest importance values (**B**,**D**); and species (**E**), genera (**F**), and family (**G**) area curves of tree communities in lowland rainforests and montane rainforests of Hainan Island. Green solid circles represent lowland rainforests, and red solid circles represent montane rainforests.

In total, 154 tree species with DBH > 3 cm were recorded in lowland rainforests, and 119 were recorded in montane rainforests, with lowland rainforests harboring 51 rare species compared to 40 rare species in montane rainforests. Within the lowland rainforest tree community, there were 1148 trees belonging to 11 dominant families (each with at least five species), accounting for 55.81% of the total number of individuals. In contrast, the montane rainforest tree community comprised 450 trees from seven dominant families (each with at least five species), which accounted for only 25.18% of the total number of individuals (Table 1). The top three families by importance value in lowland rainforests were Fagaceae, Lauraceae, and Myrtaceae. In terms of individual count, Myrtaceae, Schisandraceae, Guttiferae, and Euphorbiaceae were tied for third place. In montane rainforests, Podocarpaceae predominated in terms of both individual number and importance value (Figure 2B,D).

The results of the species–sequence curve reveal that the species-abundance distribution within the tree community conforms to a normal distribution, characterized by a longer tail (species with only one individual), which indicates a higher prevalence of rare species in both lowland and montane rainforests (Figure 2A,C). According to the data, there are 51 rare species in the 1-hectare sample plot of lowland rainforest, accounting for 33.11% of the total species and 2.46% of the total individuals. In comparison, the 1-hectare sample plot of montane rainforest contains 40 rare species, which represent 33.6% of the total tree species and 2.24% of the total individuals (Table 1).

The species–area curve does not approach smoothness, suggesting that the 1-hectare sampling area remains somewhat limited. In contrast, the genus–area curve exhibits a gentle upward trend, indicating that the 1-hectare sample is generally adequate for studying genus diversity. Meanwhile, the family-area curve tends to flatten, demonstrating that a 1-hectare sample plot is sufficient for family-level studies. Secondly, irrespective of whether we examine species, genus, or family levels, the number of species in lowland rainforests exceeds that in montane rainforests (Figure 2E–G).

### 3.2. DBH Distribution

The number of families, genera, species, and individuals in lowland rainforests exceeds that found in montane rainforests, particularly in the diameter class of 5–10 cm (Table 2). This suggests that lowland rainforests possess stronger potential for population regeneration compared to montane rainforests. The diameter class distribution of the top four dominant species in both rainforest types follows an inverted “J” shape, with the exception of the *Dacrydium pectinatum* community, which approaches a normal distribution. The relatively low number of individuals in the 5–10 cm diameter class indicates insufficient seedling replenishment, thereby complicating population renewal (Figure S2).

### 3.3. Spatial Distribution Pattern and Intraspecific Association

**Figure 3:**
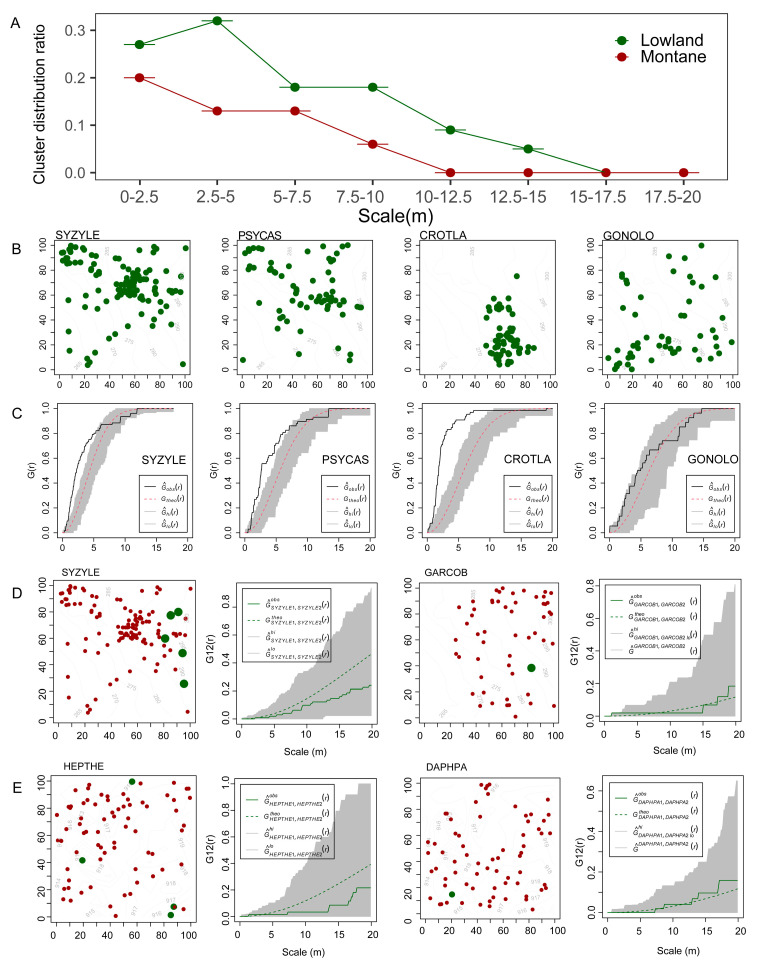
The spatial distribution of dominant species in lowland rainforests and montane rainforests. (**A**): The spatial clustering distribution ratio of dominant species in small trees; (**B**): the specific spatial location of small trees; (**C**): the corresponding point distribution pattern; (**D**,**E**): the intraspecific correlation between small and large trees (red dots: small trees with DBH ≤ 7.5 cm; green dots: large trees with DBH ≥ 25 cm). The dashed line represents the theoretical value, the solid line represents the actual observed value, and the gray area represents the upper and lower envelope lines (i.e., confidence intervals). G12(r) represents a function between two species, used to describe spatial point pattern analysis, quantifying the spatial distribution relationship between two different species or different individuals of the same species. The species codes can be found in Tables S2 and S3 (SYZYLE: *Syzygium levinei*; PSYCAS: *Psychotria asiatica*; CROTLA: *Croton laevigatus*; GONOLO: *Gonocaryum lobbianum*; GARCOB: *Garcinia oblongifolia*; HEPTHE: *Heptapleurum heptaphyllum*; DAPHPA: *Daphniphyllum paxianum*).

Based on the statistics of tree species with DBH ≤ 7.5, it is found that they have an aggregated distribution on a small scale and a random distribution on a large scale, which indicates that they are affected by the negative density dependence effect, and this effect in lowland rainforest is stronger than in montane rainforest. It can be found that the proportion of small tree clusters in lowland rainforest is higher than that in montane rainforest at all scales, especially at the scale of 2.5–5 m; the proportion of lowland rainforest cluster distribution accounts for 32% of the total, while the proportion of montane rainforest accounts for only 13% (Figure 3A). Regarding the spatial distribution pattern of the four specific dominant species within the quadrat, it can be intuitively seen that CROTLA is obviously clustered, while the other species are relatively less obvious (Figure 3B), so Figure 3C is used. It can be seen that the upper envelope lines of SYZYLE and PSYCAS are significantly higher than the lower envelope, i.e., indicating a clustered distribution (Figure 3C).

It can be seen that the number of red dots is significantly higher than that of green dots, that is, the number of small trees is higher, and there are only a few large trees, especially GARCOB and DAPHPA. There is neither a significant positive nor a significant negative correlation between large and small trees, there are no significant correlations with SYZYLE, and the same is true for GARCOB, HEPTHE, and DAPHPA (Figure 3D,E).

### 3.4. Interspecific Association

**Figure 4:**
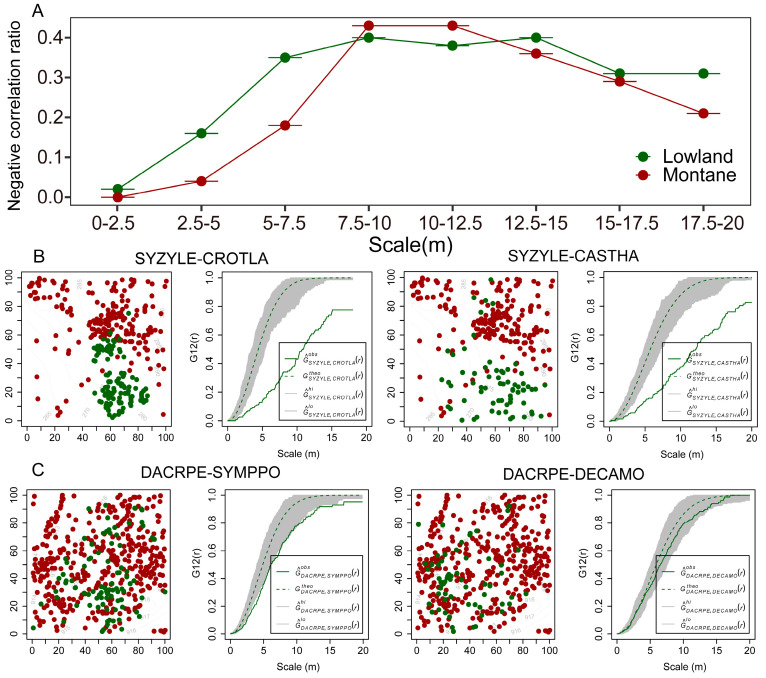
The proportion of negative correlations (**A**) and interspecific associations (**B**,**C**) between dominant species in lowland rainforests and montane rainforests (abundance ≥ 50; number = *n* × (*n* − 1)/2); lowland (n = 11); montane (n = 8)). (**B**): red dots: SYZYLE; green dots: CROTLA, CASTHA. (**C**): red dots: DACRPE; green dots: SYMPPO, DECAMO. The species codes can be found in Tables S2 and S3 (SYZYLE: *Syzygium levinei*; CROTLA: *Croton laevigatus*; CASTHA: *Castanopsis hainanensis*; DACRPE: *Dacrydium pectinatum*; SYMPPO: *Symplocos poilanei*; DECAMO: *Decaspermum montanum*;).

Our results showed that the proportion of negative correlations between interspecific associations in lowland rainforests is relatively higher than that in montane rainforests, except for two points that are slightly lower than those in montane rainforests (Figure 4A). It can be seen that the spatial distribution map of SYZYLE and CROTLA is obviously clustered separately, with red dots clustered together and green dots clustered together, and the envelope in the right figure further illustrates that SYZYLE and CROTLA are significantly negatively correlated, and similarly, the same conclusion can be drawn for SYZYLE and CASTHA. From the perspective of the spatial distribution of the montane rainforest species DACRPE, SYMPPO, and DECAMO, the red dots and green dots are mixed together, making them difficult to distinguish, and there is no obvious clustering trend. Additionally, the green solid line in the right figure also falls in the gray area, indicating that DACRPE, SYMPPO, and DECAMO have no significant correlation (Figure 4B,C).

### 3.5. Niche Differentiation and Environmental Variables

**Figure 5:**
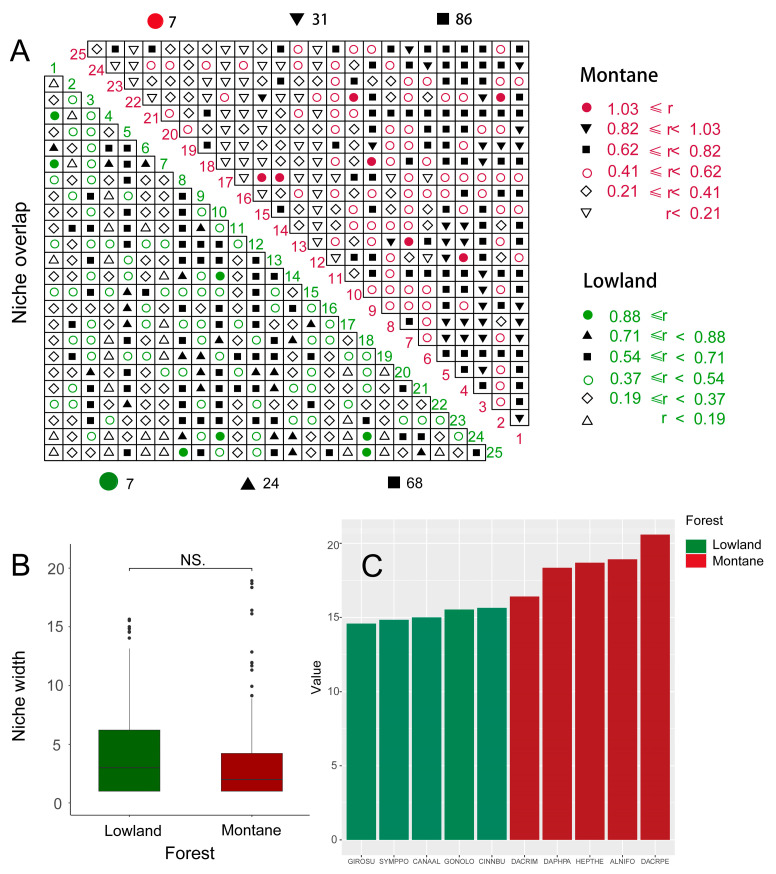
The ecological niche overlap and width between lowland rainforests and montane rainforests. (**A**): The degree of ecological niche overlap; (**B**): overall ecological niche width; (**C**): the ecological niche width of the top five tree species. The points in (B) are discrete points. These discrete points have been retained to reflect the true distribution of the data. Green represents lowland rainforests, while red represents montane rainforests. The species codes can be found in Tables S2 and S3.

**Figure 6:**
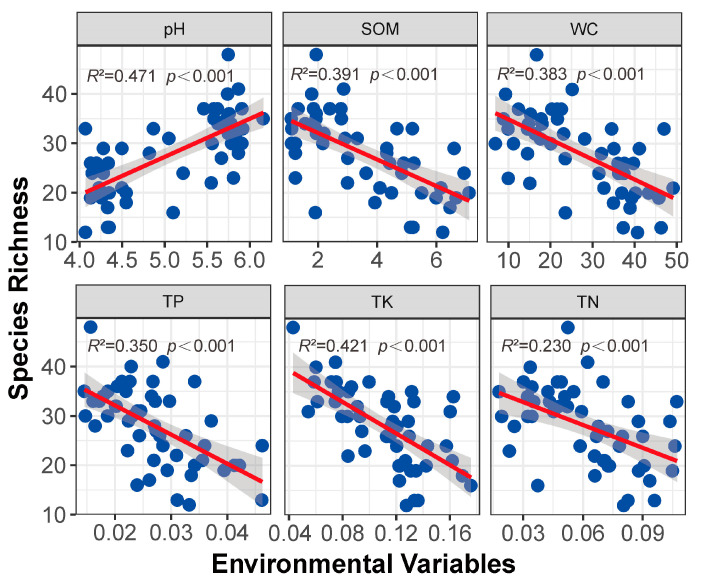
The correlation of environmental variables and species richness of lowland rainforests and montane rainforests (measurement Units: SOM: %; WC: %; TP: g/kg; TK: g/kg; TN: g/kg). The dots are data points, representing the actual observed data; the line is the regression line, which signifies the best-fitting relationship between the independent and dependent variables; the gray area indicates the confidence interval of the regression line.

From the perspective of ecological niche overlap, there are seven maximum r values of lowland rainforest and montane rainforest. The maximum r value of lowland rainforest is 0.88, and the maximum r value of montane rainforest is 1.03, indicating that the overlap degree of the ecological niches of montane rainforest is higher than those of lowland rainforest, and the second (the number for lowland rainforest is 24, the number for montane rainforest is 31) and third (the number for lowland rainforest is 68, the number for montane rainforest is 86) of the montane rainforest r values are higher than those of lowland rainforests (Figure 5A). Our results indicate that there is no significant difference in ecological niche width (*p* > 0.05) (Figure 5B,C). In other words, lowland rainforests have lower niche overlap and higher niche differentiation than montane rainforests. Upon analyzing the correlation of environmental variables and species richness of lowland rainforests and montane rainforests, we found that species richness is significantly positively correlated with pH value (*p* < 0.001) and significantly negatively correlated with soil organic matter, water content, total potassium, total phosphorus, and total nitrogen (*p* < 0.001). This means that species richness significantly increases with an increase in pH value and significantly decreases with an increase in soil nutrients (Figure 6).

## 4. Discussion

### 4.1. Tree Species Diversity

Tropical rainforests are a hot spot in biodiversity research [20,30]. In this study, a total of 154 tree species with diameters larger than 3 cm, and 119 species in montane rainforest, were recorded in lowland rainforest. Although the lowland rainforest of Diaoluo Mountain on Hainan Island has suffered from selective logging due to the historical practice of slash-and-burn agriculture, decades of recovery and the establishment of protected areas have led to our research findings indicating that its tree species diversity remains higher than that of the montane rainforest. Our results showed that the tree species diversity of lowland rainforest is higher than that of montane rainforest in terms of family and species, which is consistent with a previous study conducted in Sri Lankan rainforest, which stated that the number of tree families, genera, and species and community-scale tree diversity decreased with an increase in altitude [52]. The lowland rainforest has higher tree species diversity than the montane rainforest, which validates our first hypothesis, which can be explained by the lower elevation and higher temperature of the lowland rainforest [28]. The average annual temperature, the coldest monthly average temperature, and the hottest monthly mild temperature of the lowland rainforest are higher than those of the montane rainforest (Table S1). If global warming continues, higher temperatures may lead to an increase in species diversity in tropical montane rainforests [21]. However, this may result in a decrease in species diversity in lowland rainforests. Due to the fact that tropical rainforests already receive abundant rainfall, ample water and heat can strengthen niche overlap, thereby reducing plant diversity in lowland rainforests [53]. The species–sequence curve showed that there were more rare species in lowland rainforest and montane rainforest, and the number of rare species in lowland rainforest (51 species) was higher than that in montane rainforest (40 species). The individuals of rare species in lowland rainforest and montane rainforest on Diaoluo Mountain in Hainan account for 33.1% and 33.6% of the total species, respectively. Our study has certain limitations due to the failure to analyze potential outliers or deviations in the data. Although this proportion is lower than that of the rainforest of Xishuangbanna [19], there is still a large number of rare species in the tropical rainforest community of Diaoluo Mountain, Hainan, which has very high protection value. By comparing the climate information and soil characteristics of the two plots, we found that the climatic factors of the lowland rainforest, such as average temperature and rainfall, are generally higher than those of the montane rainforest. However, overall, the soil moisture and the content of soil organic matter, nitrogen, phosphorus, and potassium in the lowland rainforest are lower than those in the montane rainforest. Except for pH value and available soil potassium, which are higher than in the montane rainforest, all other physicochemical properties are lower than those in the montane rainforest. Although the soil texture of the lowland rainforest is poorer, its tree species diversity is still higher than that of the montane rainforest due to the stronger effects of negative density dependence and niche differentiation (Table S1).

### 4.2. Negative Density Dependence Effect

Our finding demonstrated that tree species with DBH ≤ 7.5 cm are aggregated at a small scale but randomly distributed at a large scale, which indicates that they are affected by a negative density dependence effect. Our results are in line with previous studies conducted in tropical forests which state that most of the tree species are distributed in clusters, while a small number of species are randomly distributed [41,44,54,55,56]. This is due to the limited spread distance of seeds, which cannot be spread to all the areas suitable for seed germination and growth [39,57], so that small trees gather on a small scale and their distribution is random on a large scale.

Our results also showed that the NDD effect in lowland rainforest is stronger than in montane rainforest, which confirmed our second hypothesis and is consistent with a previous study conducted in 23 old-growth temperate forest stands across a 1000 m elevation gradient [29]. LaManna et al. used temperate forest tree survival and growth data collected over more than 40 years and found that tree species diversity increased with an increase in altitude gradient and was negatively correlated with the same density [29]. Xu et al. found that the same negative density dependence decreased with an increase in species abundance [9]. This is consistent with the research results of LaManna et al., which further shows that the negative density dependence effect plays a very important role in tropical rainforest [58].

Some studies have also found that the spatial distribution pattern of the population changes with a change in scale, and the degree of aggregation usually shows a more aggregated distribution on a small scale, but with an increase in the scale, the degree of aggregation gradually weakens [59,60]. For example, a study in the Xishuangbanna *Parashorea chinensis* forest found that a reduction in the spatial aggregation of life stages is indirect evidence of the Janzen–Connell (J-C) distance effect [16]. The random distribution pattern of tree species is probably the result of fierce competition among neighboring individuals in the process of long-term ecological succession [61]. In a study on the spatial distribution characteristics of tree species in karst evergreen deciduous broad-leaved forest in southwest China, Du et al. found that the Mulun karst forest community mainly had an aggregated distribution, and the degree of aggregation and aggregation intensity decreased with an increase in spatial scale, abundance, average DBH, and maximum DBH, and the aggregation of rare species was much more than that of medium-abundance and rich species [62].

Some research results show that on a small spatial scale, competition between species for resources leads to more obvious intraspecific and interspecific relationships, affecting the distribution pattern of species [63,64]. Janzen [65] and Connell [66] believe that herbivores can reduce the number of seedlings around the mother tree and thus provide space for the survival of other species [3]. According to this hypothesis, the trees around the mother tree are randomly distributed and distributed in clusters at a certain distance from the mother tree. The negative density dependence effect of the same species of plants is a common ecological phenomenon, that is, plants have higher mortality in places where the same species gather (more pathogens, predators, etc.). The death of the same species can free up more space and resources for other species. Therefore, the negative density dependence effect can prevent a species from occupying all of the space, thus improving the coexistence of different species. Because the trees we investigated have a large DBH, lack enough data on seedlings, and have few species of big trees, there is no correlation between small trees and big trees, which can be explained by the fact that there is no obvious aggregation of small trees around big trees, which verifies the Janzen–Connell effect to some extent. Some researchers have explained nearly 90% of the changes in global species richness [67] by considering the characteristics of the climate itself and its geographical properties.

### 4.3. Niche Differentiation and Environment Variables

The study of Kang et al. confirmed that niche differentiation is an intrinsic mechanism for maintaining the relationship between community diversity and stability under grazing pressure [53]; this coincides with our research. Our research findings indicate that the degree of ecological niche overlap in lowland rainforest is lower than that in montane rainforest; in other words, lowland rainforests have more differentiated ecological niches than montane rainforests, which is more conducive to species coexistence. Alexander et al. proposed the Directed Ecological Filtration Hypothesis (DEFH), which states that species at high altitudes are generally tolerant to a wide range of environmental conditions, and that an important factor in the successful colonization of non-native species is the overlap with the tolerance range of native species [68]. Our research has also confirmed this point, determining that montane rainforests have higher altitudes, higher ecological niche overlap, and broader environmental adaptability, while lowland rainforests have lower altitudes and more severe ecological niche differentiation. In terms of niche width, the niche width of dominant species of montane rainforest, such as *Dacrydium pectinatum,* was higher than those of lowland rainforest; this is consistent with a previous study, which found that conifers had the highest niche overlap, followed by evergreen broad-leaved trees and deciduous species [69].

Dong et al. analyzed the phenological dynamics of three important alpine plants on the Qinghai–Tibet Plateau from 1997 to 2016 and found that under the background of climate warming, the overlap of phenological ecological niches between species continued to increase, with precipitation being the main influencing factor [70]. Due to sufficient precipitation in tropical rainforests, if the global climate continues to warm, in environments with favorable water and heat conditions, plants may more actively utilize nutrients and moisture in the soil, thereby leading to greater similarity in resource utilization among species. The niche width of species may increase, thus causing overlap with the niches of other species. The increasing niche overlap may lead to a decrease in plant diversity in lowland rainforests, so we need to pay attention to the impact of warming on lowland rainforests. In addition, species replacement occurs when the niche characteristics of endemic plants intersect with those of encroaching plant species [71], so we should pay attention to the species invasion of climate warming.

Maedeh et al. found, in their study of interspecific niche overlap and climate associations of native Quercus species in the Zagros Forest of Iran, that climate overlap between paired species is significant, and climate-related environmental variables, including annual precipitation and annual average temperature, are the most important driving factors for the habitat suitability of Quercus species [72]. Our plot data show that the annual precipitation and average temperature of lowland rainforests are higher than those of montane rainforests, as detailed in Table S1. This may be the main driving factor for the higher tree species diversity in lowland rainforests compared to montane rainforests. Our study also found that pH and soil nutrients have a strong correlation with species richness. The pH value is significantly positively correlated with species richness, which is consistent with the findings on microbial species richness in tropical forests [73].

Olivier and Bonaventure assessed the effects of limited dispersal and niche differentiation on the spatial distribution patterns of tree species in the tropical rainforest of Cameroon, concluding that limited dispersal is the primary factor influencing the degree of species aggregation [57]. However, our results indicate that both effects are important for the maintenance of tree species in the lowland rainforest of Hainan. Accordingly, Daisy et al. sought to integrate niche differentiation with dispersal limitation to predict succession in tropical forests [74]. They provided a better method for predicting the succession of secondary tropical forests on landscape disturbance gradients by integrating seed sources and fruit-eating-animal behavior. And uniting niche differentiation and dispersal limitation predicts tropical forest succession. Our research findings can provide data support for this study.

## 5. Conclusions

Although scientists have established several large plots for the dynamic monitoring of tropical forests to investigate the mechanisms underlying the maintenance of tree species diversity, no comparative analyses have been conducted on tree species diversity between mountain and lowland rainforests within the same region. We established 1-hectare plots in both mountain and lowland rainforest areas of Diaoluo Mountain in Hainan. Our research findings indicate that lowland rainforests have significantly higher tree species diversity than montane rainforests. More pronounced negative density-dependent effects and enhanced niche differentiation contribute to the increased tree species diversity observed in lowland rainforests. The results of this study aim to deepen our understanding of the ecological characteristics of Hainan Island’s tropical rainforests and provide a theoretical foundation for the conservation and ecological management of biodiversity in these ecosystems.

## Figures and Tables

**Table 1:** Statistics of families and species of lowland rainforest and montane rainforest in Hainan.

**Family**	**No. of Family**		**No. of Stems**	
	**Lowland**	**Montane**	**Lowland**	**Montane**
Dominant families (≥5)	11 (24.44%)	7 (19.91%)	1148 (55.81%)	450 (25.18%)
Common departments (≥2)	20 (44.44%)	16 (36.36%)	574 (27.9%)	784 (43.87%)
Single family (=1)	14 (31.11%)	21 (47.73%)	335 (16.29%)	553 (30.95%)
**Species**	**No. of Species**		**No. of Stems**	
	**Lowland**	**Montane**	**Lowland**	**Montane**
Dominant species (≥50)	11 (7.14%)	8 (6.73%)	1078 (51.90%)	1182 (66.14%)
Common species (≥2)	92 (59.7%)	71 (59.66%)	948 (45.64%)	565 (31.62%)
Rare species (=1)	51 (33.11%)	40 (33.61%)	51 (2.46%)	40 (2.24%)

**Table 2:** DBH distribution of tree species in lowland rainforest and montane rainforest.

DBH (cm)	No. of Family		No. of Genus		No. of Species		No. of Individuals	
	Lowland	Montane	Lowland	Montane	Lowland	Montane	Lowland	Montane
DBH ≥ 5	45	44	108	68	154	119	2077	1787
DBH ≥ 10	39	33	82	46	103	74	771	958
DBH ≥ 20	30	18	42	25	46	27	191	359
DBH ≥ 30	18	8	23	11	24	11	54	107
DBH ≥ 40	9	3	12	4	12	4	19	14
DBH ≥ 50	3	1	3	1	3	1	5	1

## Data Availability

Data will be made available on request.

## Supplementary Materials

The following supporting information can be downloaded at: https://www.mdpi.com/article/10.3390/plants14040505/s1, Figure S1: Diameter class distribution of dominant species in lowland rainforest (A,B) and montane rainforest (C,D). Species code can be found in Tables S2 and S3; Figure S2: Spatial distribution pattern of dominant species of small trees (DBH ≤ 7.5cm) in lowland rainfor-ests (A,B) and montane rainforests(C,D). Species code can be found in Tables S2 and S3; Figure S3: Intraspecific associations of dominant species in lowland rainforests(A,B) and montane rainfor-ests (C,D). Species code can be found in Tables S2 and S3; Figure S4: Inter species associations of dominant species in lowland rainforests (A–C) and montane rainforests (D–F). Species code can be found in Tables S2 and S3; Table S1: Plot Information of lowland rainforest and montane rainforest in Diaoluo mountain, Hainan; Table S2: Important values of tree species in the lowland rainforest of Diaoluo mountain; Table S3: Important values of tree species in the montane rainforest of Diaoluo mountain; Table S4: Important values of tree family in the lowland rainforest of Diaoluo mountain; Table S5: Important values of tree family in the montane rainforest of Diaoluo mountain; Table S6: Cluster distribution ratio in the lowland rainforest and montane rainforest of Diaoluo mountain; Table S7: Negative correlation ratio in the lowland rainforest and montane rainforest of Diaoluo mountain; Table S8: Top 25 niche width in the lowland rainforest and montane rainforest of Diaoluo mountain; Table S9: Top 25 niche overlap raw data in the lowland rainforest of Diaoluo mountain; Table S10: Top 25 niche overlap raw data in the montane rainforest of Diaoluo mountain.
