# Supplementary material for: Why Is the Diversity of Tree Species in China’s Lowland Rainforests Higher than in Its Montane Rainforests?
Source: Plants (Basel). 2025 Feb 7;14(4):505. doi: 10.3390/plants14040505 (PMC11859025; doi:10.3390/plants14040505)
Supplement: Supplementary file 1 [file plants-14-00505-s001.zip › plants-3312734-supplementary.pdf]

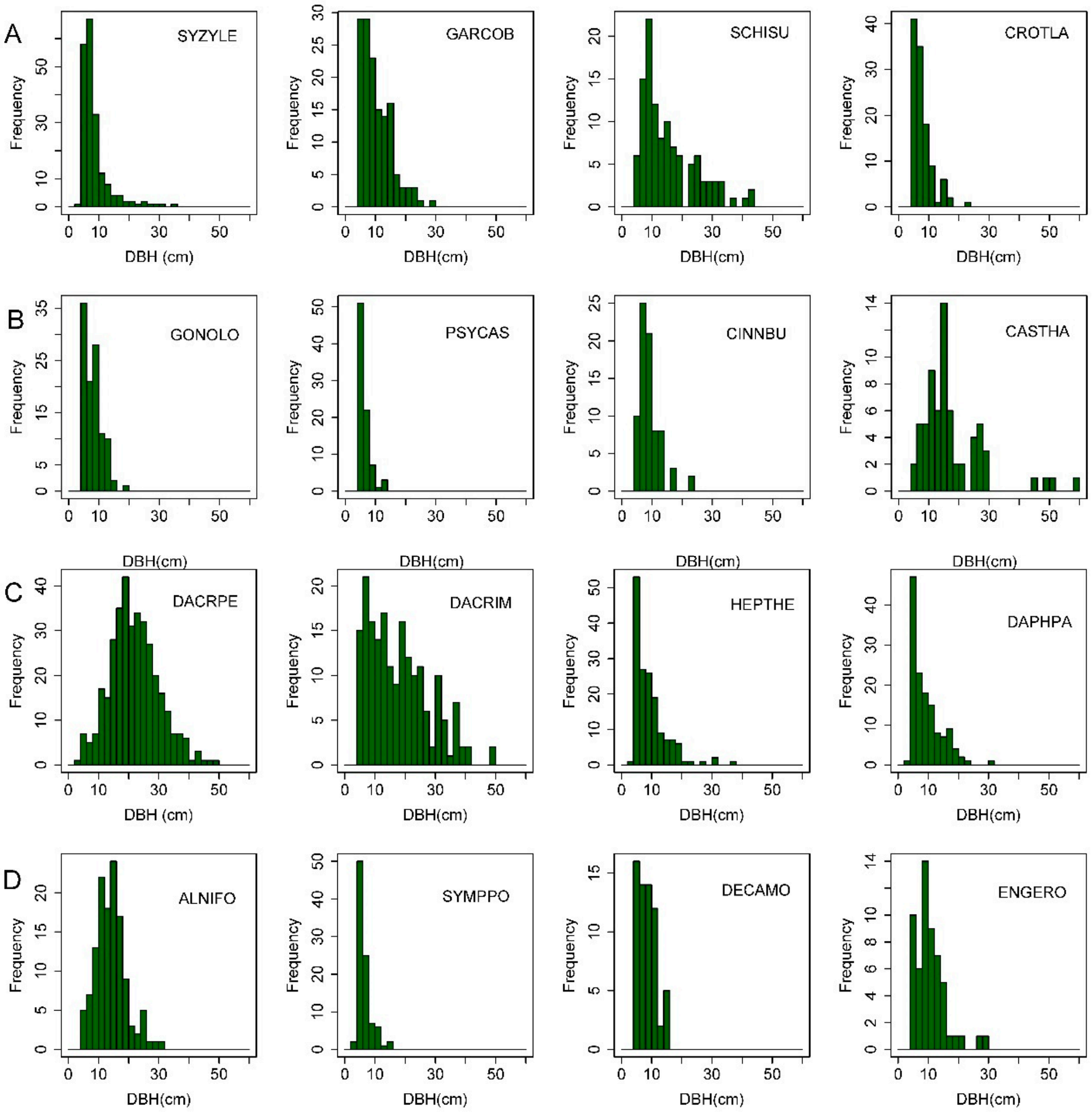

Figure S1. Diameter class distribution of dominant species in lowland rainforest (A-B) and montane rainforest (C-D). Species code can be found in Table S2 and Table S3.

 https://doi.org/10.3390/plants14040505

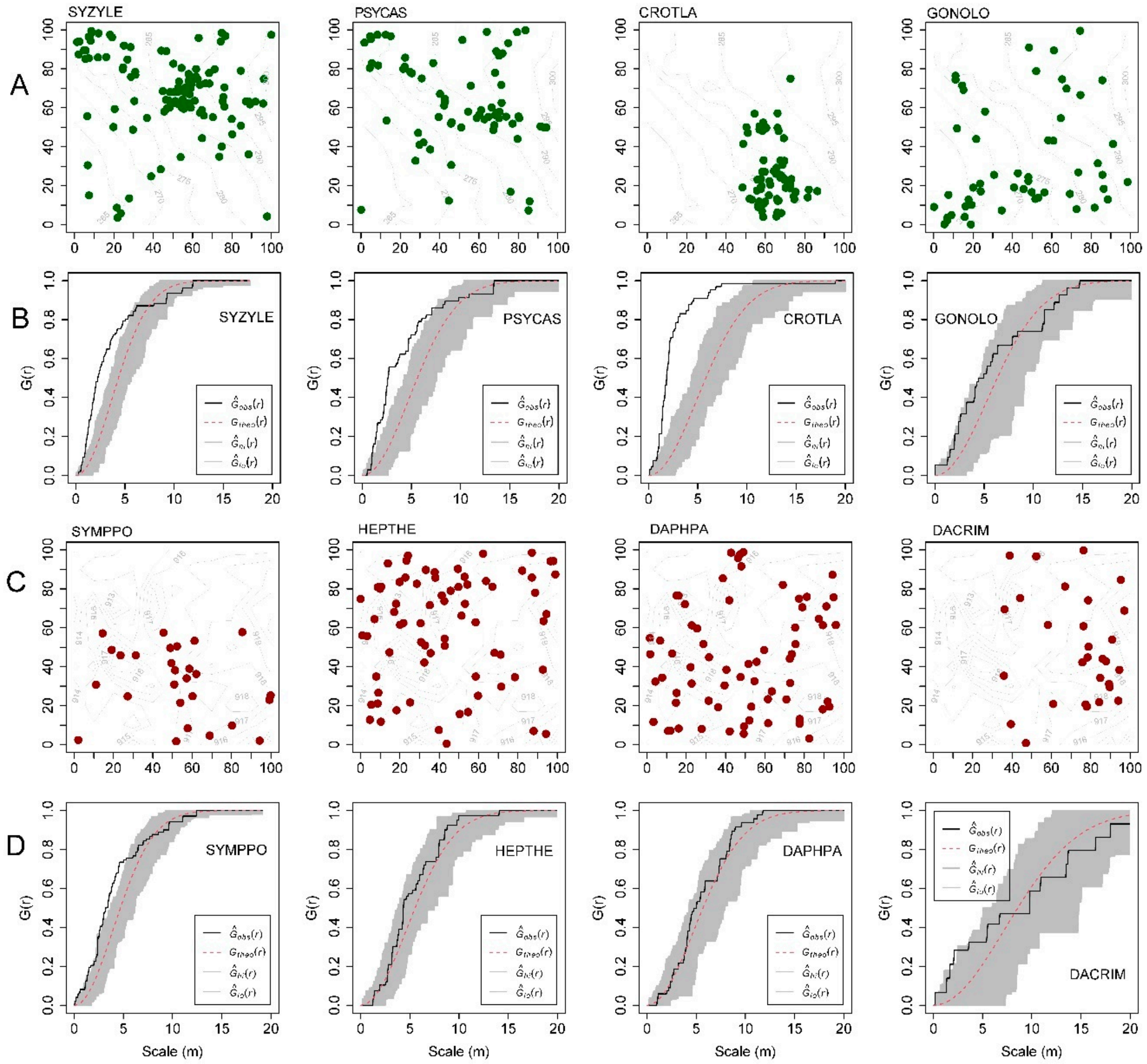

Figure S2. Spatial distribution pattern of dominant species of small trees (DBH≤7.5cm) in lowland rainforests(A&B) and montane rainforests(C&D). Species code can be found in Table S2 and Table S3.

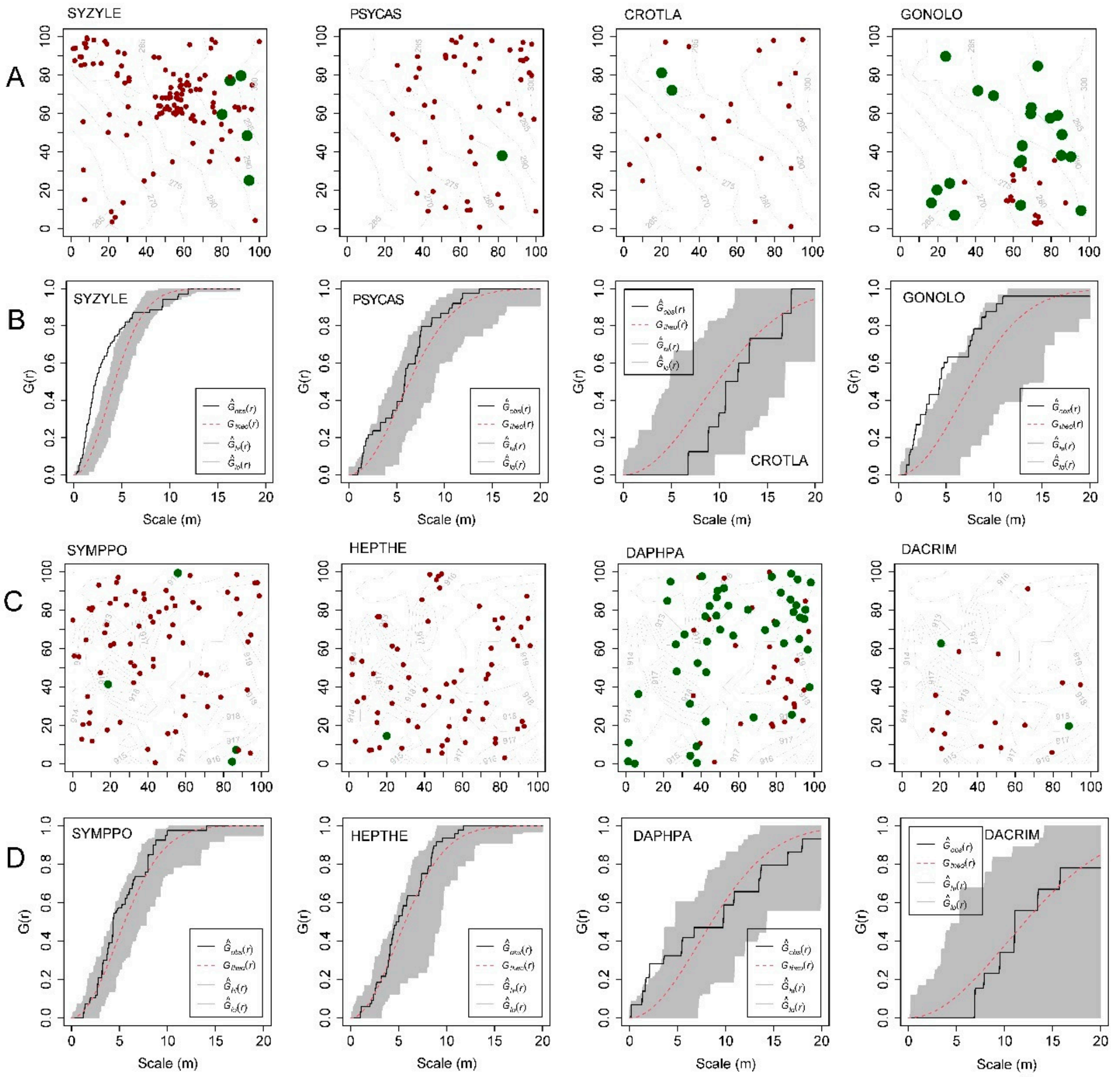

Figure S3. Intraspecific associations of dominant species in lowland rainforests(A&B) and montane rainforests(C&D). Species code can be found in Table S2 and Table S3.

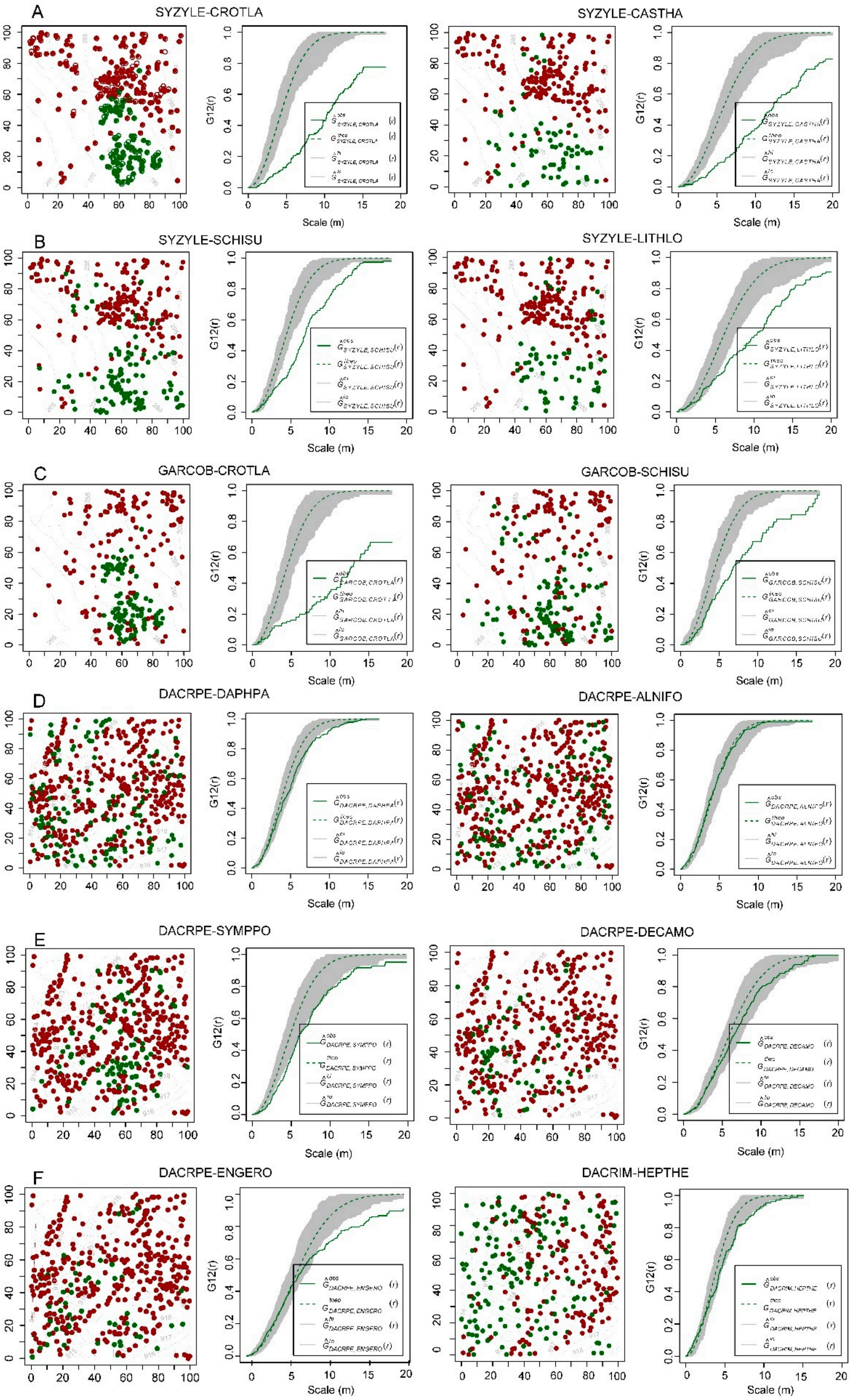

Figure S4. Inter species associations of dominant species in lowland rainforests (A-C) and montane rainforests (D-F). Species code can be found in Table S2 and Table S3.

Table S1. Plot Information of lowland rainforest and montane rainforest in Diaoluo mountain, Hainan.

Plot Information	Lowland	Montane
Latitude	18.6719198	18.7273803
Longitude	109.894289	109.867122
Altitude (m)	295	915
Annual average temperature (℃)	24.13	23.27
Average temperature of the coldest month (℃)	17.26	16.48
Average temperature of the hottest month (℃)	27.95	26.91
Annual precipitation (mm)	1526.59	1518.35
Air humidity (%)	85.33	85.76
Soil WC (%)	9.78	31.43
SOM(%)	4.54	15.34
pH	6.09	4.67
AN(mg/kg)	8.92	20.29
NN(mg/kg)	27.19	27.73
AP(mg/kg)	1.00	1.16
AK(mg/kg)	70.51	57.29
TN(g/kg)	0.11	0.78
TP(g/kg)	0.03	0.04
TK(g/kg)	0.10	0.16

Table S2. Important values of tree species in the lowland rainforest of Diaoluo mountain.

Species code	*D #R	Species	Family	Abundance	Basal area	IV
SCHISU	*#	*Schima superba*	Theaceae	113	28281.53	18.18
SYZYLE	*#	*Syzygium levinei*	Myrtaceae	198	15332.13	17.83
GARCOB	*#	*Garcinia oblongifolia*	Guttiferae	142	14703.71	15.14
CASTHA	#	*Castanopsis hainanensis*	Fagaceae	67	22335.91	12.93
GONOLO	*#	*Gonocaryum lobbianum*	Cardiopteridaceae	109	6359.36	10.59
LITHLO	*#	*Lithocarpus longipedicellatus*	Fagaceae	59	13109.58	9.24
CROTLA	*#	*Croton laevigatus*	Euphorbiaceae	113	6390.63	9
CINNBU	*#	*Cinnamomum burmanni*	Lauraceae	77	5844.38	8.26
NEOLOB	*	*Neolitsea obtusifolia*	Lauraceae	40	11082.2	8.18
CANAAL	*#	*Canarium album*	Burseraceae	60	6294.47	7.61
PSYCAS	*#	*Psychotria asiatica*	Rubiaceae	84	2887.94	7.5
SYMPPO	#	*Symplocos poilanei*	Symplocaceae	57	3882.69	6.81
GIROSU	*	*Gironniera subaequalis*	Ulmaceae	48	5397.85	6.7
PTERLA	*	*Pterospermum lanceifolium*	Malvaceae	27	7211.48	5.52
ENGERO		*Engelhardia roxburghiana*	Juglandaceae	17	8113.81	5.49
FAGASP1	*	*Fagaceae species1*	Fagaceae	33	5472.28	5.17
MELIPT		*Melicope pteleifolia*	Rutaceae	21	7286.55	4.9
RADEFR		*Radermachera frondosa*	Bignoniaceae	31	3959.75	4.87
CRATCO	*	*Cratoxylum cochinchinense*	Hypericaceae	40	3471.65	4.76
VATIMA		*Vatica mangachapoi*	Dipterocarpaceae	20	5500.16	4.55
AMESCH		*Amesiodendron chinense*	Sapindaceae	25	3069.63	4.37
LANNCO		*Lannea coromandelica*	Anacardiaceae	24	3786.45	4.23
DILLTU	*	*Dillenia turbinata*	Dilleniaceae	24	3544.45	4.02
WENDUV	*	*Wendlandia uvariifolia*	Rubiaceae	34	1564.32	3.65
TETRGL		*Tetradium glabrifolium*	Rutaceae	18	4039.35	3.44
ALSTRO		*Alstonia rostrata*	Apocynaceae	13	3370.88	3.07
KOILHA	*	*Koilodepas hainanense*	Euphorbiaceae	28	1799.13	2.97
FAGASP5		*Fagaceae species5*	Fagaceae	13	4517.15	2.89
LITSVA	*	*Litsea variabilis*	Lauraceae	20	1006.50	2.89
ELLIGL		*Ellipanthus glabrifolius*	Connaraceae	17	1418.16	2.78
SYMPCO	*	*Symplocos cochinchinensis*	Symplocaceae	14	2147.29	2.78
APORDI	*	*Aporosa dioica*	Phyllanthaceae	22	1304.19	2.74
DALBHA		*Dalbergia hainanensis*	Leguminosae	13	2178.42	2.74
TRIACO		*Triadica cochinchinensis*	Euphorbiaceae	15	1813.15	2.47
QUERPA		*Quercus patelliformis*	Fagaceae	7	3473.97	2.46
SUREMU		*Suregada multiflora*	Euphorbiaceae	16	1017.48	2.46
LITHSI		*Lithocarpus silvicolarum*	Fagaceae	19	1835.78	2.43
MICRPA	*	*Microcos paniculata*	Malvaceae	15	636.66	2.39
BACCRA	*	*Baccaurea ramiflora*	Phyllanthaceae	16	721.58	2.23
SARCLA		*Sarcosperma laurinum*	Sapotaceae	16	1568.64	2.19
SINDGL		*Sindora glabra*	Leguminosae	4	3851.01	2.1
CHIORA		*Chionanthus ramiflorus*	Oleaceae	13	804.52	1.88
TAREWA		*Tarennoidea wallichii*	Rubiaceae	11	965.85	1.84
CAMPPA		*Camphora parthenoxylon*	Lauraceae	10	642.97	1.79
MACHGA		*Machilus gamblei*	Lauraceae	4	2860.59	1.61
CELTTI		*Celtis timorensis*	Ulmaceae	9	1172.98	1.58
FAGASP2		*Fagaceae species2*	Fagaceae	9	1737.30	1.55
ANTICH		*Antirhea chinensis*	Rubiaceae	10	602.50	1.54
ACROPE		*Acronychia pedunculata*	Rutaceae	8	438.58	1.5
PHOETA		*Phoebe tavoyana*	Lauraceae	11	668.50	1.49
DIOSER		*Diospyros eriantha*	Ebenaceae	8	618.98	1.45
HEPTHE		*Heptapleurum heptaphyllum*	Araliaceae	6	1373.62	1.39

LITICH	*Litchi chinensis*	Sapindaceae	4	1903.29	1.37
FICUGL	*Ficus glaberrima*	Moraceae	6	1810.75	1.32
HOMACO	*Homalium cochinchinense*	Flacourtiaceae	8	352.78	1.23
FICUSU	*Ficus subpisocarpa*	Moraceae	7	1023.04	1.19
STEERLA	*Sterculia lanceolata*	Malvaceae	7	322.37	1.17
ELAEDU	*Elaeocarpus dubius*	Elaeocarpaceae	6	398.25	1.15
CRYPCO	*Cryptocarya concinna*	Lauraceae	7	883.05	1.14
SCOLCH	*Scolopia chinensis*	Flacourtiaceae	7	560.45	1.14
OLEADI	*Olea dioica*	Oleaceae	6	246.93	1.1
HANCHO	*Hancea hookerian*a	Euphorbiaceae	8	404.25	1.01
BRIDBA	Bridelia balansae	Phyllanthaceae	6	297.47	1
ILEXGO	*Ilex godajam*	Aquifoliaceae	5	436.81	1
ALANKU	*Alangium kurzii*	Cornaceae	5	687.94	0.97
FRAXGR	*Fraxinus griffithii*	Oleaceae	6	538.30	0.97
ALPHMO	*Alphonsea monogyna*	Annonaceae	5	165.90	0.9
VITEQU	*Vitex quinata*	Labiatae	5	184.73	0.9
APODDI	*Apodytes dimidiata*	Icacinaceae	4	579.85	0.88
DIPLDU	*Diplospora dubia*	Rubiaceae	6	250.75	0.86
unkwon		unkwon	3	840.88	0.81
HUBECE	*Huberantha cerasoides*	Annonaceae	6	375.72	0.79
ACERLA	*Acer laurinum*	Sapindaceae	4	283.28	0.77
APORVI	*Aporosa villosa*	Phyllanthaceae	4	567.15	0.76
MICRCA	*Microdesmis caseariifolia*	Pandaceae	4	157.43	0.73
POUTAN	*Pouteria annamensis*	Sapotaceae	3	621.53	0.73
ARENWE	*Arenga westerhoutii*	Palmae	1	1465.74	0.71
SYZYTE	*Syzygium tephrodes*	Myrtaceae	4	112.88	0.71
ARTOTO	*Artocarpus tonkinensis*	Moraceae	4	408.04	0.7
PELTDA	*Peltophorum dasyrrhachis*	Leguminosae	3	485.26	0.68
ILEXSP	*Ilex species1*	Aquifoliaceae	1	1269.23	0.64
HELIOB	*Helicia obovatifolia*	Proteaceae	2	798.57	0.63
ELAEPE	*Elaeocarpus petiolatus*	Elaeocarpaceae	3	326.03	0.62
SYZYCU	*Syzygium cumini*	Myrtaceae	3	330.20	0.62
GLOCSP1	*Glochidion species1*	Phyllanthaceae	3	289.34	0.61
CINNLI	*Cinnamomum liangii*	Lauraceae	3	225.42	0.59
FICUVA	*Ficus vasculosa*	Moraceae	3	186.24	0.57
FAGASP3	*Fagaceae species3*	Fagaceae	3	484.52	0.56
HELILO	*Heliciopsis lobata*	Proteaceae	3	113.65	0.54
HUNTZE	*Hunteria zeylanica*	Apocynaceae	3	88.37	0.54
NOTHOB	*Nothapodytes obtusifolia*	Icacinaceae	2	551.55	0.54
FICUBE	*Ficus benjamina*	Moraceae	2	328.80	0.46
FICUVI	*Ficus virens*	Moraceae	2	298.71	0.45
ORMOPI	*Ormosia pinnata*	Leguminosae	2	317.43	0.45
MICRFA	*Micromelum falcatum*	Rutaceae	2	200.66	0.41
GLOCSP	*Glochidion sphaerogynum*	Phyllanthaceae	2	180.24	0.4
SYZYRY	*Syzygium rysopodum*	Myrtaceae	2	177.26	0.4
BISCJA	Bischofia javanica	Phyllanthaceae	2	152.63	0.39
HERIPA	*Heritiera parvifolia*	Malvaceae	1	593.96	0.39
PRUNPH	*Prunus phaeosticta*	Rosaceae	2	138.04	0.39
TAXOIL	*Taxotrophis ilicifolia*	Moraceae	2	149.42	0.39
RHODDU	*Rhodamnia dumetorum*	Myrtaceae	2	101.36	0.37
WRIGPU	*Wrightia pubescens*	Apocynaceae	2	103.21	0.37
DECAMO	*Decaspermum montanum*	Myrtaceae	2	77.72	0.36
NEPHTO	*Nephelium topengii*	Sapindaceae	2	71.20	0.36
DIOSME	*Diospyros metcalfii*	Ebenaceae	1	263.02	0.26
MACHNA	*Machilus nanmu*	Lauraceae	1	240.53	0.26

ARDIOB	*Ardisia obtusa*	Primulaceae	2	62.02	0.24
CYCATA	*Cycas taiwaniana*	Cycadaceae	1	206.12	0.24
FICUTI	*Ficus tinctoria*	Moraceae	1	109.36	0.21
FICUMI	*Ficus microcarpa*	Moraceae	1	114.99	0.21
CRYPIM	*Cryptocarya impressinervia*	Lauraceae	1	86.59	0.2
ILEXAN	*Ilex angulata*	Aquifoliaceae	1	75.43	0.2
SYZYSP1	*Syzygium species1*	Myrtaceae	1	88.25	0.2
AIDICO	*Aidia cochinchinensis*	Rubiaceae	1	59.45	0.19
AIDIOX	*Aidia oxyodonta*	Rubiaceae	1	62.21	0.19
ALANCH	*Alangium chinense*	Cornaceae	1	69.40	0.19
BEILFO	*Beilschmiedia* fordii	Lauraceae	1	51.53	0.19
BEILIN	*Beilschmiedia intermedia*	Lauraceae	1	63.62	0.19
CRYPCH	*Cryptocarya chinensis*	Lauraceae	1	50.27	0.19
FIRMPU	*Firmiana pulcherrima*	Malvaceae	1	58.09	0.19
GLOCTR	*Glochidion triandrum*	Phyllanthaceae	1	49.02	0.19
MACLOL	*Maclurodendron oligophlebium*	Rutaceae	1	72.38	0.19
MEMEHA	*Memecylon hainanense*	Melastomataceae	1	58.09	0.19
MEMESC	*Memecylon scutellatum*	Melastomataceae	1	59.45	0.19
POLYSP	*Polyalthia species1*	Annonaceae	1	49.02	0.19
REEVBO	*Reevesia botingensis*	Malvaceae	1	50.27	0.19
SYZYST	*Syzygium stenocladum*	Myrtaceae	1	56.75	0.19
ARTOHY	*Artocarpus hypargyreus*	Moraceae	1	28.27	0.18
BEILBR	*Beilschmiedia brevipaniculata*	Lauraceae	1	28.27	0.18
DILLIN	*Dillenia indica*	Dilleniaceae	1	24.63	0.18
DIOSLO	*Diospyros longibracteata*	Ebenaceae	1	23.76	0.18
FAGASP4	*Fagaceae species4*	Fagaceae	1	20.43	0.18
FICUFI	*Ficus fistulosa*	Moraceae	1	23.76	0.18
ILEXPU	*Ilex pubilimba*	Aquifoliaceae	1	40.72	0.18
ILEXRO	*Ilex rotunda*	Aquifoliaceae	1	22.90	0.18
MALLPA	*Mallotus paniculatus*	Euphorbiaceae	1	38.48	0.18
MALLPE	*Mallotus peltatus*	Euphorbiaceae	1	39.59	0.18
MELASA	*Melastoma sanguineum*	Melastomataceae	1	20.43	0.18
MILIHO	*Miliusa horsfieldii*	Annonaceae	1	37.39	0.18
MUSABA	*Musa basjoo*	Musaceae	1	38.48	0.18
PHYLSP1	*Phyllanthus species1*	Phyllanthaceae	1	39.59	0.18
PYGETO	*Pygeum topengii*	Rosaceae	1	32.17	0.18
RADEHA	*Radermachera hainanensis*	Bignoniaceae	1	45.36	0.18
SYMPLA	*Symplocos lancifolia*	Symplocaceae	1	26.42	0.18
XANTHA	*Xanthophyllum hainanense*	Polygalaceae	1	37.39	0.18
ALLOLA	*Allospondias lakonensis*	Anacardiaceae	1	18.86	0.17
BREYFR	*Breynia fruticosa*	Phyllanthaceae	1	19.63	0.17
BRIDTO	*Bridelia tomentosa*	Phyllanthaceae	1	15.90	0.17
MACHCH	*Machilus chinensis*	Lauraceae	1	19.63	0.17
MILIBA	*Miliusa balansae*	Annonaceae	1	15.21	0.17
SYZYAR	*Syzygium araiocladum*	Myrtaceae	1	19.63	0.17
SYZYHA	*Syzygium hancei*	Myrtaceae	1	19.63	0.17

(*Distribution pattern; #Interspecific relationship).

 https://doi.org/10.3390/plants14040505

Table S3. Important values of tree species in the montane rainforest of Diaoluo mountain.

Species code	*D #R	Species	Family	Abundance	Basal area (cm²)	IV
DACRPE	*#	*Dacrydium pectinatum*	Podocarpaceae	356	153783.80	67.65
DACRIM	*#	*Dacrycarpus imbricatus*	Podocarpaceae	189	62550.85	32.27
ALNIFO	*#	*Alniphyllum fortunei*	Styracaceae	128	22609.62	17.8
HEPTHE	*#	*Heptapleurum heptaphyllum*	Araliaceae	161	15439.09	17.62
DAPHPA	*#	*Daphniphyllum paxianum*	Daphniphyllaceae	136	11850.67	15.39
SYMPPO	*#	*Symplocos poilanei*	Symplocaceae	93	3497.84	9.75
ENGERO	*#	*Engelhardia roxburghiana*	Juglandaceae	56	6098.95	7.88
DECAMO	*#	*Decaspermum montanum*	Myrtaceae	63	3989.08	7.85
POLYHA		*Polyspora hainanensis*	Theaceae	37	6469.86	7.45
ADINHA	*	*Adinandra hainanensis*	Pentaphylacaceae	27	3175.63	5.07
FRANLO	*	*Frangula longipes*	Rhamnaceae	31	1166.52	4.55
PINUCA		*Pinus caribaea*	Pinaceae	11	9294.10	4.48
SYMPLA	*	*Symplocos lancifolia*	Symplocaceae	29	2212.48	4.38
SARCLA	*	*Sarcosperma laurinum*	Sapotaceae	21	1796.57	4.35
FAGA		Fagaceae	Fagaceae	19	3437.70	3.63
MACHGA		*Machilus gamblei*	Lauraceae	15	3811.50	3.34
ARCHCL	*	*Archidendron clypearia*	Fabaceae	29	1312.52	3.24
FICUVA	*	*Ficus variolosa*	Moraceae	16	833.43	3.09
SYMPSP1	*	*Symplocos species1*	Symplocaceae	18	782.38	3.01
PENTEU		*Pentaphylax euryoides*	Pentaphylacaceae	13	1128.77	3
LINDKW		*Lindera kwangtungensis*	Lauraceae	14	1426.46	2.96
CASTFA		*Castanopsis faberi*	Fagaceae	9	5805.71	2.85
MANGFO		*Manglietia fordiana*	Magnoliaceae	13	1121.89	2.82
SYMPPS		*Symplocos pseudobarberina*	Symplocaceae	15	967.89	2.71
TOXISU		*Toxicodendron succedaneum*	Anacardiaceae	12	1109.67	2.59
LITHLI		*Lithocarpus litseifolius*	Fagaceae	11	758.32	2.25
AITICH		*Altingia chinensis*	Aitingiaceae	11	3850.27	2.23
ACERFA		*Acer fabri*	Aceraceae	10	750.46	2.02
LAUR		Lauraceae	Lauraceae	11	1111.16	1.82
CAMEOL		*Camellia oleifera*	Theaceae	10	645.24	1.81
GLOCTR		*Glochidion triandrum*	Phyllanthaceae	7	553.64	1.79
TURPMO		*Turpinia montana*	Staphyleaceae	9	460.28	1.7
LITHHA		*Lithocarpus handelianus*	Fagaceae	7	1858.42	1.45
EURY		*Eurya*	Pentaphylacaceae	8	257.12	1.41
SYZYAR		*Syzygium araiocladum*	Myrtaceae	7	134.55	1.32
GYMNPO		*Gymnosphaera podophylla*	Cyatheaceae	5	1097.71	1.3
MELIAN		*Meliosma angustifolia*	Sabiaceae	5	222.50	1.23
LIQUFO		*Liquidambar formosana*	Aitingiaceae	4	2154.81	1.19
SYZY		*Syzygium*	Myrtaceae	7	139.86	1.14
ARDICR		*Ardisia crenata*	Primulaceae	6	192.60	1.1
FICUFI		*Ficus fistulosa*	Moraceae	5	316.54	1.08
TRIACO		*Triadica cochinchinensis*	Euphorbiaceae	4	1115.18	1.07
HELILO		*Helicia longipetiolata*	Proteaceae	5	255.36	1.06
AITIOB		*Altingia obovata*	Aitingiaceae	3	1228.50	1.05
ALSOSP		*Alsophila spinulosa*	Cyatheaceae	4	326.31	1.03
CUNNLA		*Cunninghamia lanceolata*	Cupressaceae	9	488.17	1
EURYCU		*Eurya cuneata*	Pentaphylacaceae	4	231.99	1
POLYCA		*Polyosma cambodiana*	Escalloniaceae	4	125.77	0.97
SCHISU		*Schima superba*	Theaceae	4	608.34	0.93
ADINHO		*Adinandra howii*	Pentaphylacaceae	4	581.08	0.92
ARCHLU		*Archidendron lucidum*	Fabaceae	5	872.60	0.88
MICHSH		*Michelia shiluensis*	Magnoliaceae	3	502.64	0.84

 https://doi.org/10.3390/plants14040505

SPHABR	*Sphaeropteris brunoniana*	Cyatheaceae	3	502.79	0.84
FICUOL	*Ficus oligodon*	Moraceae	4	257.15	0.83
GLOCSP1	*Glochidion species1*	Phyllanthaceae	3	329.98	0.79
SYZYSP1	*Syzygium species1*	Myrtaceae	3	61.49	0.72
ILEXCH	*Ilex chapaensis*	Aquifoliaceae	2	681.49	0.66
ELAESP1	*Elaeocarpus species1*	Elaeocarpaceae	2	396.63	0.58
SYZYBU	*Syzygium buxifolioideum*	Myrtaceae	2	380.96	0.58
EURYCI	*Eurya ciliata*	Pentaphylacaceae	2	369.49	0.57
FICUTU	*Ficus tuphapensis*	Moraceae	3	144.08	0.56
XANTHA	*Xanthophyllum hainanense*	Polygalaceae	3	135.87	0.56
ILEXSP1	*Ilex species1*	Aquifoliaceae	3	78.88	0.55
HELIHA	*Helicia hainanensis*	Proteaceae	2	263.22	0.54
MELISP1	*Meliaceae species1*	Meliaceae	2	249.29	0.54
RHAPIN	*Rhaphiolepis indica*	Rosaceae	2	239.91	0.54
SYZYFL	*Syzygium fluviatile*	Myrtaceae	2	142.82	0.51
CLEYOB	*Cleyera obscurinervia*	Pentaphylacaceae	2	85.05	0.49
SYZYTE	*Syzygium tetragonum*	Myrtaceae	2	61.46	0.49
ANNEFR	*Anneslea fragrans*	Pentaphylacaceae	2	46.97	0.48
CRATCO	*Cratoxylum cochinchinense*	Hypericaceae	2	49.80	0.48
GLOCWR	*Glochidion wrightii*	Phyllanthaceae	2	45.80	0.48
ILEXFI	*Ilex ficoidea*	Aquifoliaceae	2	40.87	0.48
CYCLSP1	*Cyclobalanopsis species1*	Fagaceae	1	804.25	0.46
ENGELE	*Engelhardtia Lesch*	Juglandaceae	3	218.98	0.41
TETRGL	*Tetradium glabrifolium*	Rutaceae	3	100.33	0.37
LITHSI	*Lithocarpus silvicolarum*	Fagaceae	2	256.77	0.36
FICUAU	*Ficus auriculata*	Moraceae	2	153.47	0.33
ILEXTR	*Ilex triflora*	Aquifoliaceae	1	320.47	0.32
ZANTAV	*Zanthoxylum avicennae*	Rutaceae	2	121.15	0.32
ANTIMA	*Antidesma maclurei*	Phyllanthaceae	2	83.04	0.31
ELAEDU	*Elaeocarpus dubius*	Elaeocarpaceae	1	271.72	0.31
ILEXHA	*Ilex hainanensis*	Aquifoliaceae	1	188.69	0.29
EHRELO	*Ehretia longiflora*	Boraginaceae	1	111.22	0.27
FAGASP2	*Fagaceae species2*	Fagaceae	1	124.69	0.27
LINDNA	*Lindera nacusua*	Lauraceae	1	114.99	0.27
WENDUV	*Wendlandia uvariifolia*	Rubiaceae	1	134.78	0.27
LITHLO	*Lithocarpus longipedicellatus*	Fagaceae	1	86.59	0.26
LITHSP1	*Lithocarpus species1*	Fagaceae	1	83.32	0.26
MELISP2	*Meliaceae species2*	Meliaceae	1	78.54	0.26
THEA	Theaceae	Theaceae	1	84.95	0.26
ARTOST	*Artocarpus styracifolius*	Moraceae	1	58.09	0.25
CAMPPA	*Camphora parthenoxylon*	Lauraceae	1	63.62	0.25
ELAESP	*Elaeocarpaceae species*	Elaeocarpaceae	1	45.36	0.25
FAGASP1	*Fagaceae species1*	Fagaceae	1	58.09	0.25
GARCMU	*Garcinia multiflora*	Clusiaceae	1	54.11	0.25
ILEXLI	*Ilex liangii*	Aquifoliaceae	1	50.27	0.25
LITHEL	*Lithocarpus elaeagnifolius*	Fagaceae	1	69.40	0.25
LLEXSU	*Ilex subficoidea*	Aquifoliaceae	1	41.85	0.25
VERNCU	*Vernonia cumingiana*	Asteraceae	1	63.62	0.25
ILEXLI	*Ilex liangii*	Aquifoliaceae	1	19.63	0.24
DISEPL	*Disepalum plagioneurum*	Annonaceae	1	19.63	0.24
ELAEJA	*Elaeocarpus japonicus*	Elaeocarpaceae	1	13.20	0.24
EUONLA	*Euonymus laxiflorus*	Celastraceae	1	19.63	0.24
EUONSP1	*Euonymus species1*	Celastraceae	1	22.06	0.24
EURYSP1	*Eurya species1*	Pentaphylacaceae	1	19.63	0.24
EURYSP2	*Eurya species2*	Pentaphylacaceae	1	23.76	0.24

ILEXCO	*Ilex cochinchinensis*	Aquifoliaceae	1	19.63	0.24
LIRIAL	*Lirianthe albosericea*	Magnoliaceae	1	18.86	0.24
LITSSP1	*Lauraceae species1*	Lauraceae	1	23.76	0.24
MELAMA	*Melastoma malabathricum*	Melastomataceae	1	19.63	0.24
MORERU	*Morella rubra*	Myricaceae	1	21.24	0.24
MYRSLI	*Myrsine linearis*	Primulaceae	1	31.17	0.24
PSYDDI	*Psydrax dicocca*	Rubiaceae	1	20.43	0.24
RHODTO	*Rhodomyrtus tomentosa*	Myrtaceae	1	26.42	0.24
STROES	*Strobocalyx esculenta*	Asteraceae	1	30.19	0.24
SYMP	Symplocaceae	Symplocaceae	1	24.63	0.24
SYMPOV	*Symplocos ovatilobata*	Symplocaceae	1	18.86	0.24
SYZYSP2	*Syzygium species2*	Myrtaceae	1	19.63	0.24

(*Distribution pattern; #Interspecific relationship).

Table S4. Important values of tree family in the lowland rainforest of Diaoluo mountain.

Rank	Family	No. of species	Abundance	Basal area (cm^2)	IV
1	Fagaceae	9	210	52908.76	37.54
2	Lauraceae	15	180	23812.77	27.49
3	Myrtaceae	10	215	15760.07	20.90
4	Guttiferae	2	182	18189.49	19.98
5	Theaceae	1	114	28305.55	18.34
6	Euphorbiaceae	7	182	11225.96	18.22
7	Rubiaceae	8	150	6682.36	16.41
8	Cardiopteridaceae	1	109	6359.36	10.61
9	Rutaceae	5	49	11985.99	10.30
10	Malvaceae	6	52	8872.82	9.88
11	Symplocaceae	3	72	6056.40	9.79
12	Ulmaceae	2	57	6570.83	8.31
13	Phyllanthaceae	10	56	3347.41	8.25
14	Burseraceae	1	59	6194.18	7.55
15	Sapindaceae	4	35	5327.40	6.90
16	Leguminosae	4	22	6832.12	6.00
17	Moraceae	11	31	4202.45	5.81
18	Bignoniaceae	2	32	4345.98	5.20
19	Juglandaceae	1	17	7049.36	5.13
20	Dipterocarpaceae	1	20	5499.04	4.57
21	Anacardiaceae	2	25	3446.23	4.28
22	Dilleniaceae	2	25	3569.09	4.21
23	Apocynaceae	3	18	3562.46	3.99
24	Oleaceae	3	25	1589.75	3.95
25	Sapotaceae	2	19	2190.17	2.93
26	Connaraceae	1	17	1090.18	2.66
27	Flacourtiaceae	2	15	913.23	2.38
28	Annonaceae	5	14	643.23	2.23
29	Aquifoliaceae	5	9	1845.10	2.20
30	Ebenaceae	3	10	905.76	1.90
31	Elaeocarpaceae	2	9	724.29	1.78
32	Icacinaceae	2	6	1131.40	1.43
33	Araliaceae	1	6	1373.62	1.40
34	Proteaceae	2	5	912.22	1.18
35	Cornaceae	2	6	757.34	1.17
36	Labiatae	2	5	184.73	0.91
37	unkwon	1	3	840.88	0.82
38	Pandaceae	1	4	157.43	0.73
39	Palmae	1	1	1465.74	0.72
40	Rosaceae	2	3	170.21	0.57
41	Melastomataceae	3	3	137.96	0.55
42	Cycadaceae	1	1	206.12	0.24
43	Primulaceae	1	2	62.02	0.24
44	Musaceae	1	1	38.48	0.18
45	Polygalaceae	1	1	37.39	0.18

 https://doi.org/10.3390/plants14040505

Table S5. Important values of tree family in the montane rainforest of Diaoluo mountain.

Rank	Family	No. of species	Abundance	Basal area (cm^2)	IV
1	Podocarpaceae	2	545	216334.65	99.92
2	Symplocaceae	6	157	7504.08	20.33
3	Styracaceae	1	128	22609.62	17.8
4	Araliaceae	1	161	15439.09	17.62
5	Daphniphyllaceae	1	136	11850.67	15.39
6	Pentaphylacaceae	10	64	5919.51	13.43
7	Myrtaceae	9	88	4956.26	13.08
8	Fagaceae	11	54	13343.26	12.29
9	Theaceae	4	52	7808.38	10.45
10	Lauraceae	6	43	6551.49	8.88
11	Juglandaceae	2	59	6317.93	8.28
12	Moraceae	6	31	1762.76	6.15
13	Rhamnaceae	1	31	1166.52	4.55
14	Pinaceae	1	11	9294.10	4.48
15	Aitingiaceae	3	18	7233.57	4.47
16	Sapotaceae	1	21	1796.57	4.35
17	Fabaceae	2	34	2185.12	4.12
18	Magnoliaceae	3	17	1643.38	3.9
19	Phyllanthaceae	4	14	1012.46	3.38
20	Aquifoliaceae	9	13	1441.79	3.27
21	Cyatheaceae	3	12	1926.81	3.17
22	Anacardiaceae	1	12	1109.67	2.59
23	Sapindaceae	1	10	750.46	2.02
24	Staphyleaceae	1	9	460.28	1.7
25	Proteaceae	2	7	518.58	1.61
26	Elaeocarpaceae	4	5	726.91	1.37
27	Primulaceae	2	7	223.77	1.34
28	Sabiaceae	1	5	222.50	1.23
29	Euphorbiaceae	1	4	1115.18	1.07
30	Cupressaceae	1	9	488.17	1
31	Escalloniaceae	1	4	125.77	0.97
32	Meliaceae	2	3	327.83	0.79
33	Rutaceae	2	5	221.47	0.7
34	Polygalaceae	1	3	135.87	0.56
35	Rosaceae	1	2	239.91	0.54
36	Rubiaceae	2	2	155.21	0.51
37	Asteraceae	2	2	93.81	0.49
38	Hypericaceae	1	2	49.80	0.48
39	Celastraceae	2	2	41.70	0.48
40	Boraginaceae	1	1	111.22	0.27
41	Clusiaceae	1	1	54.11	0.25
42	Myricaceae	1	1	21.24	0.24
43	Annonaceae	1	1	19.63	0.24
44	Melastomataceae	1	1	19.63	0.24

Table S6. Cluster distribution ratio in the lowland rainforest and montane rainforest of Diaoluo mountain.

Lowland	0-2.5m	2.5-5m	5-7.5m	7.5-10m	10-12.5m	12.5-15m	15-17.5m	17.5-20m
SYZYLE	Cluster	Cluster	Random	Random	Random	Random	Random	Random
PSYCAS	Cluster	Cluster	Random	Random	Random	Random	Random	Random
CROTLA	Cluster	Cluster	Cluster	Cluster	Random	Random	Random	Random
GONOLO	Random	Random	Random	Random	Random	Random	Random	Random
GARCOB	Random	Random	Random	Random	Random	Random	Random	Random
CINNBU	Random	Cluster	Random	Random	Random	Random	Random	Random
SYMPCO	Random	Random	Random	Random	Random	Random	Random	Random
WENDUV	Random	Random	Random	Random	Random	Random	Random	Random
GIROSU	Random	Random	Random	Random	Random	Random	Random	Random
CANAAL	Random	Random	Random	Random	Random	Random	Random	Random
SCHISU	Cluster	Cluster	Cluster	Cluster	Random	Random	Random	Random
LITHLO	Random	Random	Cluster	Cluster	Cluster	Random	Random	Random
KOILHA	Cluster	Cluster	Cluster	Cluster	Cluster	Cluster	Random	Random
CRATCO	Random	Cluster	Random	Random	Random	Random	Random	Random
PTERLA	Random	Random	Random	Random	Random	Random	Random	Random
FAGASP1	Random	Random	Random	Random	Random	Random	Random	Random
DILLTU	Cluster	Random	Random	Random	Random	Random	Random	Random
LITSVA	Random	Random	Random	Random	Random	Random	Random	Random
BACCRA	Random	Random	Random	Random	Random	Random	Random	Random
NEOLOB	Random	Random	Random	Random	Random	Random	Random	Random
APORDI	Random	Random	Random	Random	Random	Random	Random	Random
MICRPA	Random	Random	Random	Random	Random	Random	Random	Random
Cluster ratio	6/22	7/22	4/22	4/22	2/22	1/22	0	0
Montane	0-2.5m	2.5-5m	5-7.5m	7.5-10m	10-12.5m	12.5-15m	15-17.5m	17.5-20m
SYMPPO	Cluster	Cluster	Random	Random	Random	Random	Random	Random
HEPTHE	Random	Random	Random	Random	Random	Random	Random	Random
DAPHPA	Random	Random	Random	Random	Random	Random	Random	Random
DACRIM	Cluster	Random	Random	Random	Random	Random	Random	Random
DECAMO	Random	Random	Cluster	Random	Random	Random	Random	Random
ARCHCL	Cluster	Cluster	Cluster	Cluster	Random	Random	Random	Random
FRANLO	Random	Random	Random	Random	Random	Random	Random	Random
ENGERO	Random	Random	Random	Random	Random	Random	Random	Random
DACRPE	Random	Random	Random	Random	Random	Random	Random	Random
SYMPLA	Random	Random	Random	Random	Random	Random	Random	Random
ADINHA	Random	Random	Random	Random	Random	Random	Random	Random
ALNIFO	Random	Random	Random	Random	Random	Random	Random	Random
FICUVA	Random	Random	Random	Random	Random	Random	Random	Random
SARCLA	Random	Random	Random	Random	Random	Random	Random	Random
SYMPSP1	Random	Random	Random	Random	Random	Random	Random	Random
Cluster ratio	3/15	2/15	2/15	1/15	0	0	0	0

Table S7. Negative correlation ratio in the lowland rainforest and montane rainforest of Diaoluo mountain.

Lowland	0-2.5m	2.5-5m	5-7.5m	7.5-10m	10-12.5m	12.5-15m	15-17.5m	17.5-20m
SYZYLE-GARCOB	No	No	No	No	No	No	No	No
SYZYLE-CROTLA	Negative	Negative	Negative	Negative	Negative	Negative	Negative	Negative
SYZYLE-SCHISU	No	Negative	Negative	Negative	Negative	Negative	No	No
SYZYLE-GONOLO	No	No	No	No	No	No	No	No
SYZYLE-PSYCAS	Positive	No	No	No	No	No	No	No
SYZYLE-CINNBU	No	No	Negative	Negative	Negative	Negative	No	No
SYZYLE-CASTHA	No	Negative	Negative	Negative	Negative	Negative	Negative	Negative
SYZYLE-CANAAL	No	No	No	No	No	No	No	No
SYZYLE-LITHLO	No	No	Negative	Negative	Negative	Negative	Negative	Negative
SYZYLE-SYMPPO	No	No	No	No	No	No	No	No
GARCOB-CROTLA	No	Negative	Negative	Negative	Negative	Negative	Negative	Negative
GARCOB-SCHISU	No	No	Negative	Negative	Negative	Negative	Negative	Negative
GARCOB-GONOLO	No	Negative	No	No	No	No	No	No
GARCOB-PSYCAS	No	No	No	Negative	No	No	No	No
GARCOB-CINNBU	No	No	No	Negative	Negative	Negative	Negative	Negative
GARCOB-CASTHA	No	No	Negative	Negative	Negative	Negative	Negative	Negative
GARCOB-CANAAL	No	No	No	No	No	No	No	No
GARCOB-LITHLO	No	No	Negative	Negative	Negative	Negative	Negative	Negative
GARCOB-SYMPPO	No	No	No	No	No	Negative	Negative	Negative
CROTLA-SCHISU	Positive	Positive	No	No	No	No	No	No
CROTLA-GONOLO	No	Negative	No	No	No	No	No	No
CROTLA-PSYCAS	No	No	Negative	Negative	Negative	Negative	No	No
CROTLA-CINNBU	No	No	No	No	No	No	No	No
CROTLA-CASTHA	No	Positive	No	No	No	No	No	No
CROTLA-CANAAL	No	No	No	No	No	No	No	No
CROTLA-LITHLO	No	No	No	No	No	No	No	No
CROTLA-SYMPPO	No	Positive	Positive	Positive	No	No	No	No
SCHISU-GONOLO	No	No	No	No	No	No	No	No
SCHISU-PSYCAS	No	Negative	Negative	Negative	Negative	Negative	No	No
SCHISU-CINNBU	No	No	No	No	No	No	No	No
SCHISU-CASTHA	No	Positive	No	No	No	No	No	No
SCHISU-CANAAL	No	No	No	No	No	No	No	No
SCHISU-LITHLO	Positive	No	No	No	No	No	Negative	Negative
SCHISU-SYMPPO	No	No	No	No	No	No	No	No
GONOLO-PSYCAS	No	No	Negative	Negative	Negative	Negative	No	No
GONOLO-CINNBU	No	No	Negative	Negative	Negative	Negative	No	No
GONOLO-CASTHA	No	No	Negative	Negative	Negative	Negative	Negative	Negative
GONOLO-CANAAL	No	No	No	No	No	No	No	No
GONOLO-LITHLO	No	No	Negative	Negative	Negative	Negative	Negative	Negative
GONOLO-SYMPPO	No	No	No	No	No	No	No	No
PSYCAS-CINNBU	No	No	No	No	No	No	No	No
PSYCAS-CASTHA	No	Negative	Negative	Negative	Negative	Negative	Negative	Negative
PSYCAS-CANAAL	No	No	No	No	No	No	No	No
PSYCAS-LITHLO	No	Negative	Negative	Negative	Negative	Negative	Negative	Negative
PSYCAS-SYMPPO	No	No	No	No	No	No	No	No
CINNBU-CASTHA	No	No	No	Negative	Negative	Negative	Negative	Negative
CINNBU-CANAAL	No	No	No	No	No	No	No	No
CINNBU-LITHLO	No	No	Negative	Negative	Negative	Negative	Negative	Negative
CINNBU-SYMPPO	No	No	No	No	No	No	No	No
CASTHA-CANAAL	No	No	No	No	No	No	No	No
CASTHA-LITHLO	No	Positive	No	No	No	No	No	No

CASTHA-SYMPPO	No	No	No	No	No	No	No	No
CANAAL-LITHLO	No	No	Negative	Negative	Negative	Negative	Negative	Negative
CANAAL-SYMPPO	No	No	No	No	No	No	No	No
LITHLO-SYMPPO	No	No	No	No	No	No	No	No
Negative	1	9	19	22	21	22	17	17
Positive	3	5	1	1	0	0	0	0
No	51	41	35	32	34	33	38	38
Negative correlation ratio	1/55	9/55	19/55	22/55	21/55	22/55	17/55	17/55
Montane	0-2.5m	2.5-5m	5-7.5m	7.5-10m	10-12.5m	12.5-15m	15-17.5m	17.5-20m
DACRPE-DACRIM	No	No	Negative	Negative	Negative	No	No	No
DACRPE-HEPTHE	No	No	No	Negative	Negative	No	No	No
DACRPE-DAPHPA	No	No	No	Negative	No	No	No	No
DACRPE-ALNIFO	No	No	No	No	No	No	No	No
DACRPE-SYMPPO	No	Negative	No	No	Negative	Negative	Negative	Negative
DACRPE-DECAMO	No	No	No	No	No	No	No	No
DACRPE-ENGERO	No	No	No	Negative	Negative	Negative	Negative	Negative
DACRIM-HEPTHE	No	No	No	No	No	No	No	No
DACRIM-DAPHPA	No	No	Negative	Negative	No	No	No	No
DACRIM-ALNIFO	No	No	No	No	No	No	No	No
DACRIM-SYMPPO	No	No	No	Negative	Negative	Negative	No	No
DACRIM-DECAMO	No	No	Negative	Negative	Negative	No	No	No
DACRIM-ENGERO	No	No	No	Negative	Negative	Negative	Negative	Negative
HEPTHE-DAPHPA	No	No	No	No	No	No	No	No
HEPTHE-ALNIFO	No	No	No	No	Negative	No	No	No
HEPTHE-SYMPPO	No	No	Negative	Negative	Negative	Negative	No	No
HEPTHE-DECAMO	No	No	No	No	No	No	No	No
HEPTHE-ENGERO	No	No	No	No	No	Negative	Negative	No
DAPHPA-ALNIFO	No	No	No	No	No	No	No	No
DAPHPA-SYMPPO	No	No	No	Negative	Negative	Negative	Negative	Negative
DAPHPA-DECAMO	No	No	No	No	No	No	No	No
DAPHPA-ENGERO	No	No	No	Negative	Negative	Negative	Negative	Negative
ALNIFO-SYMPPO	No	No	Negative	Negative	Negative	Negative	Negative	Negative
ALNIFO-DECAMO	No	No	No	No	No	No	No	No
ALNIFO-ENGERO	No	No	No	No	No	Negative	Negative	No
SYMPPO-DECAMO	No	No	No	No	No	No	No	No
SYMPPO-ENGERO	No	Positive	No	No	No	No	No	No
DECAMO-ENGERO	No	Positive	No	No	No	No	No	No
Negative	0	1	5	12	12	10	8	6
Positive	0	2	0	0	0	0	0	0
No	28	25	23	16	16	18	20	22
Negative correlation ratio	0	1/28	5/28	12/28	12/28	10/28	8/28	6/28

Table S8. Top 25 niche width in the lowland rainforest and montane rainforest of Diaoluo mountain.

	Lowland		Montane	
	Species	Width	Species	Width
1	CINNBU	15.64	DACRPE	20.59
2	GONOLO	15.53	ALNIFO	18.92
3	CANAAL	15.00	HEPTHE	18.69
4	SYMPPO	14.84	DAPHPA	18.35
5	GIROSU	14.58	DACRIM	16.41
6	AMESCH	14.53	POLYHA	16.11
7	NEOLOB	14.04	ENGERO	12.85
8	GARCOB	13.14	ADINHA	11.95
9	ENGERO	12.57	DECAMO	11.92
10	RADEFR	12.48	SYMPPO	11.67
11	LANNCO	11.08	SARCLA	11.31
12	MICRPA	10.71	PENTEU	9.94
13	LITSVA	10.53	FRANLO	9.15
14	PTERLA	10.27	FICUVA	9.14
15	VATIMA	10.00	LINDKW	8.91
16	FAGASP1	9.99	SYMPLA	8.33
17	ELLIGL	9.97	SYMPPS	7.76
18	DALBHA	9.94	SYMPSP1	7.71
19	PSYCAS	9.88	TOXISU	7.20
20	SYZYLE	9.60	GLOCTR	7.00
21	DILLTU	9.29	MANGFO	6.76
22	SUREMU	9.14	LITHLI	6.37
23	SYMPCO	8.91	FAGASP1	6.12
24	SCHISU	8.70	ACERFA	5.56
25	LITHLO	8.43	MACHGA	5.49
26	Average	11.55	Average	10.97

plants

MDPI

Table S9. Top 25 niche overlap raw data in the lowland rainforest of Diaoluo mountain.

Species	SCHISU	SYZYLE	GARCOB	CASTHA	GONOLO	LITHLO	CROTLA	CINNBU	NEOLOB	CANAAL	PSYCAS	SYMPPO	GIROSU	PTERLA	ENGERO	FAGASP	MELIPT	RADEFR	CRATCO	VATIMA	AMESCH	LANNCO	DILTU	WENDUV	TETRGL
plot1	1	1	1	0	16	0	0	2	0	4	1	2	1	0	1	5	3	1	0	0	0	1	0	0	0
plot2	4	4	1	4	5	0	0	3	1	7	0	1	0	2	0	0	1	1	0	0	0	4	0	2	0
plot3	20	0	8	4	8	6	18	7	1	4	1	3	0	2	1	1	0	1	0	1	0	3	2	0	0
plot4	22	0	8	13	4	5	23	3	0	0	1	3	1	0	2	1	0	0	0	1	1	2	0	0	0
plot5	13	1	4	4	5	6	8	5	1	3	2	3	1	1	0	3	0	0	0	1	0	1	0	0	0
plot6	2	1	2	0	1	0	0	3	0	5	0	1	5	3	1	1	0	4	0	0	0	0	2	5	2
plot7	2	1	3	4	3	0	0	7	1	0	2	2	3	0	1	2	1	0	1	0	1	1	0	1	3
plot8	12	2	2	7	3	13	11	1	0	2	1	5	1	2	2	4	0	2	3	1	1	0	1	0	0
plot9	13	1	4	17	1	7	19	5	1	2	0	5	1	0	1	0	2	0	1	0	0	0	3	0	0
plot10	4	4	3	4	5	8	2	0	0	3	0	3	1	2	1	4	4	2	0	0	0	2	0	0	0
plot11	0	2	0	0	2	0	0	6	1	1	1	3	5	2	0	0	0	0	1	0	1	0	0	4	1
plot12	0	4	7	3	4	0	0	7	1	3	4	3	1	5	0	4	0	4	2	0	2	0	2	3	3
plot13	2	6	4	2	2	2	21	3	4	4	5	9	5	0	0	0	0	1	1	3	3	0	1	2	1
plot14	5	11	6	0	6	0	9	4	4	1	19	2	0	0	0	0	1	0	5	1	2	1	0	0	1
plot15	2	12	3	0	2	4	0	6	3	3	5	2	2	1	1	0	2	1	13	0	1	1	1	1	0
plot16	0	0	1	0	5	0	0	5	4	1	0	0	0	2	0	3	0	3	0	0	1	0	1	2	5
plot17	4	8	2	0	1	0	0	4	0	6	5	1	2	3	0	0	0	3	0	3	2	0	1	9	1
plot18	2	42	7	1	2	2	1	0	3	2	6	1	1	0	1	0	3	2	2	0	1	2	0	1	0
plot19	1	31	5	0	11	1	1	3	0	0	6	1	0	0	2	0	2	2	2	0	1	1	0	0	1
plot20	0	12	7	0	4	2	0	1	2	2	1	2	3	0	0	1	0	0	6	1	1	0	1	0	0
plot21	0	21	1	0	5	0	0	0	2	0	10	2	2	0	0	3	0	0	0	1	2	0	0	0	0
plot22	2	7	8	0	2	0	0	1	2	1	5	1	4	1	0	1	0	1	0	1	1	1	3	0	0
plot23	1	6	15	4	1	1	0	0	3	0	1	0	2	0	1	0	0	1	1	1	0	2	5	3	0
plot24	1	15	17	0	3	2	0	1	4	5	7	2	3	1	1	0	2	2	0	2	2	0	1	1	0
plot25	0	6	23	0	8	0	0	0	2	1	1	0	4	0	1	0	0	0	2	3	2	2	0	0	0

Table S10. Top 25 niche overlap raw data in the montane rainforest of Diaoluo mountain.

Species	DA<br>CR<br>PE	DA<br>CRI<br>M	AL<br>NIF<br>O	HE<br>PT<br>HE	DA<br>PH<br>PA	SY<br>MP<br>PO	EN<br>GE<br>RO	DE<br>CA<br>MO	PO<br>LY<br>HA	AD<br>IN<br>HA	FR<br>AN<br>LO	PI<br>NU<br>CA	SY<br>MP<br>LA	SA<br>RC<br>LA	FA<br>GA<br>SP	MA<br>CH<br>GA	AR<br>CH<br>CL	FIC<br>UV<br>A	SY<br>MP<br>SP1	PE<br>NT<br>EU	LI<br>ND<br>KW	CA<br>STF<br>A	MA<br>NG<br>FO	SY<br>MP<br>PS	TO<br>XI<br>SU
plot1	11	5	7	13	8	1	7	2	1	0	1	0	4	1	2	0	1	1	1	0	0	0	1	1	1
plot2	8	6	12	6	8	1	2	4	3	2	0	1	0	1	1	1	0	2	1	0	0	0	1	0	0
plot3	13	5	12	6	12	7	5	4	3	0	6	1	1	1	1	0	0	1	3	1	0	1	0	1	0
plot4	12	7	6	1	6	7	3	1	3	1	2	0	1	0	3	0	1	1	0	0	2	1	0	0	1
plot5	5	1	2	5	5	1	1	0	2	0	0	0	0	4	5	0	14	0	0	0	0	0	1	0	1
plot6	18	2	6	11	14	4	4	9	0	2	0	1	5	1	0	0	0	0	0	0	1	0	0	0	0
plot7	17	4	7	3	5	7	5	8	3	1	2	0	4	0	0	0	0	2	0	1	2	0	0	3	1
plot8	11	8	4	5	5	18	2	4	1	2	2	0	1	0	1	1	0	0	2	2	1	0	0	2	1
plot9	8	9	5	5	7	10	3	3	0	1	5	0	2	1	0	0	0	0	1	0	1	0	4	0	3
plot10	12	17	6	2	1	2	2	1	1	4	1	0	0	0	4	1	9	0	1	0	0	5	1	0	0
plot11	29	0	6	10	7	0	3	6	1	3	0	0	0	1	0	0	2	0	0	0	0	0	1	0	1
plot12	16	2	3	13	6	4	7	7	1	1	1	0	3	2	0	0	1	1	0	1	2	0	0	0	2
plot13	11	8	2	7	5	5	5	1	0	0	3	0	5	1	0	5	0	1	0	1	1	0	1	2	0
plot14	20	13	2	2	5	5	0	2	2	0	1	0	0	0	0	0	0	2	4	1	1	0	0	1	0
plot15	32	19	4	0	1	0	2	0	3	0	0	0	0	0	0	0	0	0	1	1	0	0	0	0	0
plot16	12	1	9	6	5	0	0	2	0	3	0	1	0	2	0	0	0	0	0	0	0	0	1	0	0
plot17	9	3	3	7	2	1	1	5	3	1	0	2	0	1	0	2	0	1	0	1	0	0	0	0	0
plot18	14	9	4	13	1	6	0	1	1	1	1	0	0	0	0	2	0	0	0	0	0	0	0	2	0
plot19	22	15	5	4	4	4	0	1	1	0	1	0	1	0	0	2	0	0	0	0	0	0	0	2	0
plot20	15	14	7	4	9	3	0	0	1	2	0	0	0	1	0	0	1	0	0	0	1	0	0	1	0
plot21	9	0	4	7	5	0	0	0	0	1	0	1	0	1	1	0	0	0	0	2	0	0	1	0	0
plot22	9	7	3	13	5	0	0	0	1	2	0	4	1	0	1	0	0	0	0	1	0	2	1	0	0
plot23	9	14	0	7	8	3	1	0	1	0	0	0	1	2	0	1	0	0	0	0	0	0	0	0	0
plot24	24	6	2	5	1	1	3	2	2	0	4	0	0	1	0	0	0	1	2	1	0	0	0	0	1
plot25	10	14	7	6	1	3	0	0	3	0	1	0	0	0	0	0	0	3	2	0	2	0	0	0	0

 https://doi.org/10.3390/plants14040505
